# GFAT1 promotes the progression of hepatocellular carcinoma via enhancing the *O*-GlcNAcylation of VEZF1

**DOI:** 10.1038/s41419-025-07975-5

**Published:** 2025-08-26

**Authors:** Jia-yao Yang, Rong Zhang, Zhi-rong Zhang, Shan Li, De-ao Gong, Chen-hao Li, Chang Chen, Lu-yi Huang, Ai-long Huang, Ni Tang, Kai Wang

**Affiliations:** 1 Key Laboratory of Molecular Biology for Infectious Diseases (Ministry of Education), Institute for Viral Hepatitis, Department of Infectious Diseases, The Second Affiliated Hospital, Chongqing Medical University, Chongqing, China; 2 College of Pharmacy, Chongqing Medical University, Chongqing, China

**Keywords:** Liver cancer, Liver cancer

## Abstract

Glutamine-fructose-6-phosphate amidotransferase 1 (GFAT1), the first rate-limiting enzyme in the hexosamine biosynthetic pathway (HBP), is a pivotal regulator of HBP flux. Despite its established significance, the molecular underpinnings of GFAT1’s role in hepatocellular carcinoma (HCC) remain to be elucidated. Here, we found that GFAT1 was upregulated in HCC, and high GFAT1 level was correlated with poor patient prognosis. Our in vitro and in vivo studies demonstrated that GFAT1 facilitated hepatoma cell proliferation and invasion by enhancing HBP and *O-*GlcNAcylation through its enzymatic activity. Global profiling of *O-*GlcNAcylation identified vascular endothelial zinc finger protein 1 (VEZF1) as a key substrate heavily *O-*GlcNAcylated in GFAT1-overexpressing hepatoma cells. Notably, *O-*GlcNAcylation at specific serine residues (Ser123 and Ser124) within VEZF1 attenuated its proteasomal degradation, thereby enhancing its protein stability and promoting *tensin 1* (*TNS1*) transcription in HCC. In addition, we designed a bioactive VEZF1-derived peptide to competitively inhibit GFAT1-mediated *O-*GlcNAcylation of VEZF1. This intervention effectively reduced TNS1 expression and suppressed the progression of HCC in a mouse model. Collectively, our findings underscore the therapeutic potential of targeting the GFAT1-VEZF1-TNS1 signaling axis in HCC.

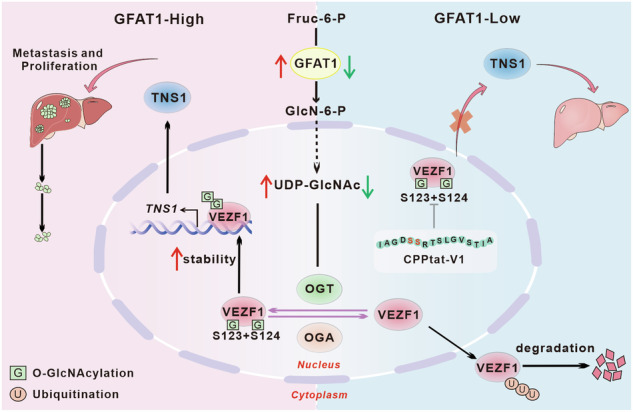

## Introduction

Hepatocellular carcinoma (HCC) is one of the most common malignant tumors globally. According to the 2020 global cancer burden data released by the International Agency for Research on Cancer (IARC), the incidence of liver cancer ranks sixth among all malignancies, with its mortality rate holding the third among all cancers [1]. The liver, as the principal metabolic organ in human body, is pivotal in maintaining homeostasis through its involvement in the metabolism of carbohydrates, proteins, amino acids and lipids. Metabolic remodeling is an important feature of cancer, characterized by metabolic alterations in tumor cell to support their survival and growth [2]. The Warburg effect shows that even under aerobic conditions, the energy metabolism of tumor cells is mainly limited to glycolysis, in which the intermediate products will be used to synthesize biological macromolecules that fuels for tumor growth [3]. Emerging evidence suggests that cancer cells undergo more complex metabolic reprogramming to meet the energy and material demands of their rapid proliferation [4].

In the context of glucose metabolism, a fraction of fructose-6 phosphate (Fru-6-P) is diverted from glycolysis into the hexosamine biosynthesis pathway (HBP), with an estimated 2–5% contribution [5]. Glucose, fatty acids, amino acids and glutamine, as vital nutrients for tumor growth, serve as substrates for HBP, facilitating the biosynthesis of uridine diphosphate N-acetylglucosamine (UDP-GlcNAc) [6]. UDP-GlcNAc is a donor substrate for *O-*linked β-N-acetylglucosamine modification (*O-*GlcNAcylation). Accumulating evidence indicates that HBP flux and protein *O-*GlcNAcylation levels are generally elevated in tumor cells [7], suggesting that the HBP plays an important role in tumor progression [8].

Glutamine-fructose-6-phosphate amidotransferase 1 (GFAT1), the first rate-limiting enzyme in the HBP, is a key regulator of HBP flux. Recent studies have underscored the multifaceted role of GFAT1 in tumorigenesis. GFAT1 is overexpressed in pancreatic cancer stem cells, with its downregulation leading to diminished tumorigenicity both in vitro and in vivo [9]. Elevated GFAT1 expression in breast cancer correlates with short disease-free survival, particularly in aggressive forms of the disease [10]. In lung cancer cells, the abnormal expression of GFAT1 is closely related to the survival and proliferation of tumor cells [11, 12]. Furthermore, the increased expression of GFAT1, induced by high glucose, has been shown to promote the migration and invasion of cholangiocarcinoma cells [13]. Additionally, GFAT1’s enzymatic activity has been implicated in increasing HBP flux in bladder cancer vascular endothelial cells, enhancing seryl-tRNA synthetase (SerRS) *O-*GlcNAcylation, inhibiting its nuclear translocation, and subsequently promoting tumor angiogenesis [14]. In HCC, FOXA2 transcriptionally activate GFAT1 expression, conferring resistance to doxorubicin-induced apoptosis, with high GFAT1 levels being associated with poor prognosis of HCC [15, 16]. However, the precise molecular mechanisms by which GFAT1 overexpression promotes HCC progression remain to be elucidated.

Vascular endothelial zinc finger 1 (VEZF1, also known as DB1 or ZNF161) is a Krüppel-like zinc finger protein belonging to the zinc finger protein family, the largest family of transcription factors within the human genome, which characterized by six Cys2/His2-type zinc finger motifs, a glutamine-stretch region, and a proline-rich transcriptional transactivation domain [17]. VEZF1 has been implicated in the transcriptional activation of SETBP1, thereby promoting ovarian cancer progression [18]. VEZF1 is also highly expressed in HCC, with its overexpression facilitating the proliferation and metastasis of HCC cells. VEZF1 transcriptionally activates progestin and adipoQ receptor 4 (PAQR4) to accelerate HCC progression, while STUB1-mediated ubiquitination of VEZF1 leads to diminished transcriptional activity and attenuated liver cancer development [19]. However, the role of *O-*GlcNAcylation in modulating VEZF1 function is far from explored.

In this study, we delve into the role of GFAT1 as a key factor in controlling HBP flux. Our results reveal that GFAT1 promotes tumor proliferation and migration by enhancing the *O-*GlcNAcylation levels in HCC. Here, we report that VEZF1 undergoes *O-*GlcNAcylation as identified by proteomics. VEZF1 is stabilized by GFAT1-mediated *O-*GlcNAcylation, thereby enhancing the binding of VEZF1 to the promoter region of *Tensin 1* (*TNS1*), activating the transcription of *TNS1*. Our findings reveal that the GFAT1-VEZF1-TNS1 axis plays a key role in regulating HCC progression, and suggest new therapeutic strategies.

## Results

### GFAT1 is upregulated in HCC and correlates with dismal patient survival

**Fig. 1:**
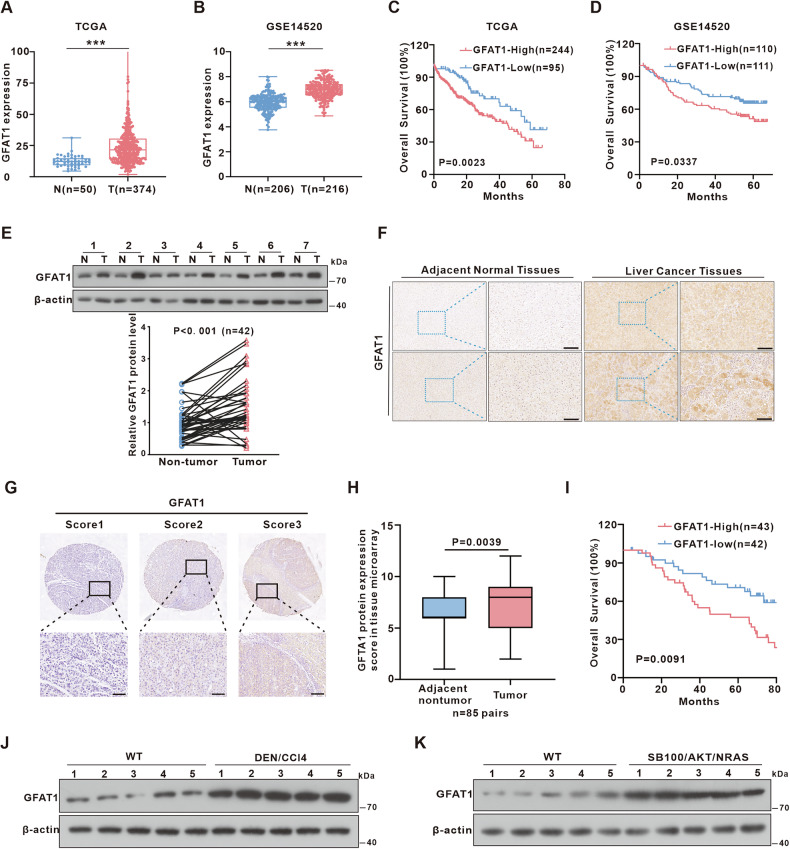
Expression and prognostic value of GFAT1 in hepatocellular carcinoma. **A,B** Analysis of mRNA expression level of *GFAT1* in HCC and noncancerous tissues based on GEO (GSE14520) and TCGA database. **C,D** Kaplan–Meier survival analysis of *GFAT1* depicting the overall survival (OS) of patients with HCC from the TCGA and GEO (GSE14520) database. **E** Western blot was used to detect the protein expression of GFAT1 in 42 pairs of HCC tissues (T) and the adjacent non-tumor tissues (N) of HCC patients, the expression levels were quantified by Image J and analyzed with two-tailed paired t-test. ***P < 0.001 (also see Fig. S1C). **F** Immunohistochemistry (IHC) staining of GFAT1 in human HCC and adjacent non-tumor tissues. Scale bar: 100 μm. **G** Representative IHC staining of GFAT1 in an HCC tissue microarray. Scores (0–3) were calculated according to the percentage of stained cells and intensity. Scale bar: 80 μm. **H** Staining scores of GFAT1 in tumor and paracancerous tissues from patients with HCC. **I** Kaplan–Meier analysis of overall survival of GFAT1 in the tissue microarray cohort (*n* = 85), HCC patients were grouped according to the GFAT1 expression level in tumor. **J,K** Immunoblots analysis of GFAT1 in normal mouse (wild-type, WT) liver tissues, DEN/CCl_4_-induced HCC tissues, and hydrodynamic tail vein injection of transposase-based plasmids (SB100/AKT/NRAS)-induced HCC tissues.

Given the proposed role of the HBP-mediated *O-*GlcNAc modification in HCC, we investigated the expression and prognostic relevance of enzymes in HBP. First, we compared the mRNA levels of *glutamine-fructose-6-phosphate aminotransferase 1* (*GFAT1*), *glucosamine-6-phosphate N-acetyltransferase 1* (*GNPNAT1*), *phosphoglucomutase 3* (*PGM3*), and *UDP-N-acetylglucosamine pyrophosphorylase 1* (*UAP1*) in The Cancer Genome Atlas-Liver Hepatocellular Carcinoma [TCGA-LIHC]) dataset [20] and GEO (GSE14520) dataset [21]. The mRNA expression of *GFAT1* was significantly upregulated in HCC tissues, whereas *GNPNAT1*, *PGM3* and *UAP1* only showed a slight increase or decrease (Fig. 1A, B and Fig. S1A). Importantly, the high mRNA expression of *GFAT1* was significantly correlated with poor overall survival in patients with HCC (Fig. 1C, D and Fig. S1B). Subsequently, GFAT1 protein expression was assessed in 42 pairs of clinical HCC tissues and adjacent non-tumor tissues by Western blot. The result demonstrated that the protein levels of GFAT1 in HCC tissues were significantly higher than the non-tumorous counterparts (Fig. 1E). Moreover, immunohistochemistry (IHC) staining of clinical HCC and adjacent non-tumor tissue slices confirmed these findings (Fig. 1F). To further analyze the relationship between GFAT1 expression and survival in clinical HCC tissues, we performed immunohistochemical analyses of HCC patients using tissue microarrays. As shown in Fig. 1G, patients were stratified according to the level of GFAT1. The results showed that the expression level of GFAT1 protein was higher in liver cancer tissues compared with adjacent tissues, and the high expression of GFAT1 was significantly associated with poor overall survival (Fig. 1H, I). Additionally, GFAT1 was highly expressed in mouse model of HCC induced by diethylnitrosamine (DEN) and carbon tetrachloride (CCl_4_), as well as in a mouse model utilizing hydrodynamic tail vein injection of Sleeping Beauty (SB) transposase-based plasmids expressing SB100/AKT/NRAS (Fig. 1J, K).

### GFAT1 promotes hepatoma cell proliferation and invasion by enhancing the HBP and *O-*GlcNAcylation

**Fig. 2:**
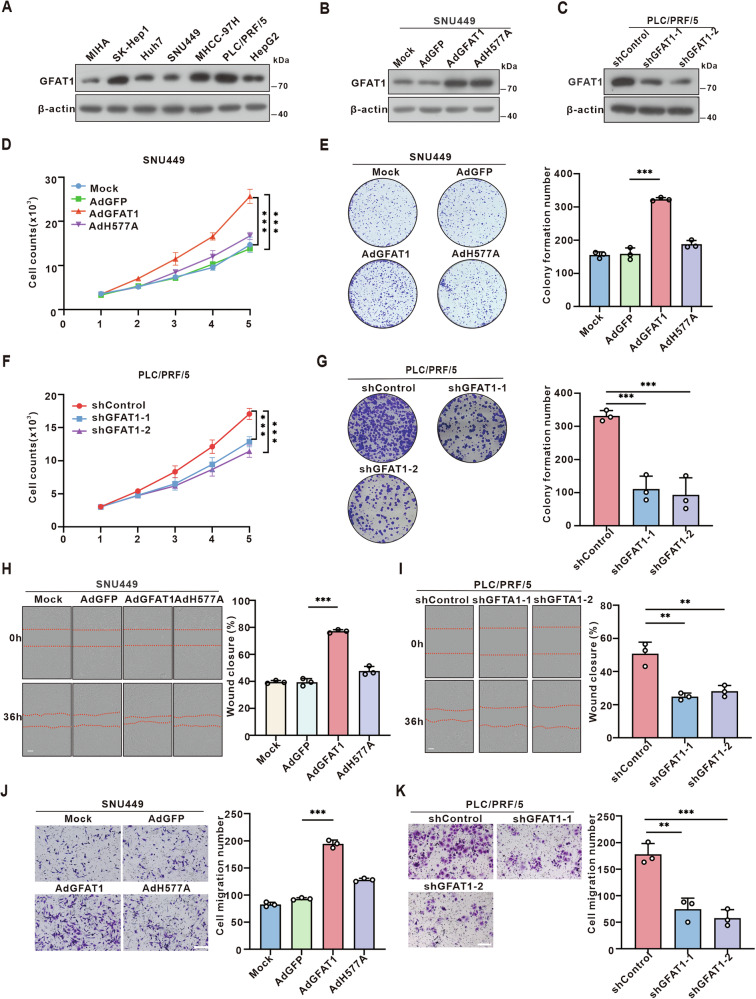
GFAT1 promotes hepatoma cell proliferation and tumorigenesis. **A** The protein level of GFAT1 in HCC cell lines was tested by Western blotting. **B** Western blot was used to detect the protein expression of GFAT1 in HCC cells infected with AdGFAT1-WT, GFAT1-H577A mutant adenovirus **C** or shGFAT1 lentivirus. **D, F** Growth curves of hepatoma cells. **E, G** Colony formation capacity of SNU449 and PLC/PRF/5 cells. **H–K** Representative and quantified results of the wound-healing assays **(H,I)** and transwell **(J,K)** in SNU449 or in PLC/PRF/5 cells. Statistical analysis was shown as mean ± SD (*n* = 3). One-way ANOVA followed by the Tukey test, *P < 0.05, **P < 0.01, ***P < 0.001. Scale bar: 50 μm **(H,I)**, 20 μm **(J, K)**.

To explore the role of GFAT1 in the proliferation and invasion of hepatoma cells, we firstly examined the intrinsic expression of GFAT1 in various HCC cell lines (Fig. 2A). Subsequently, we utilized the AdEasy adenovirus system to overexpress wild-type (WT) GFAT1 and its enzyme-inactivating mutant H577A (Fig. 2B and Fig. S2A). Growth curves and clone formation assays were conducted in SUN449 and Huh7 cells, which exhibit relatively low endogenous GFAT1 levels. The results indicated that GFAT1-WT significantly promoted the proliferation of HCC cells, whereas the GFAT1-H577A mutant, lacking enzymatic activity, did not exert a similar effect (Fig. 2D, E and Fig. S2B, C). This implies that the ability of GFAT1 to promote the proliferation of HCC cells is dependent on its enzymatic activity. In contrast, knockdown of GFAT1 by lentivirus-encoded shRNA in PLC/PRF/5 cells resulted in a marked reduction in cell proliferation and colony formation (Fig. 2C, F, G). Additionally, transwell and wound-healing assays showed that the promotion of HCC cell invasion and migration by GFAT1 was also dependent on its enzymatic activity (Fig. 2H–K and Fig. S2D, E).

**Fig. 3:**
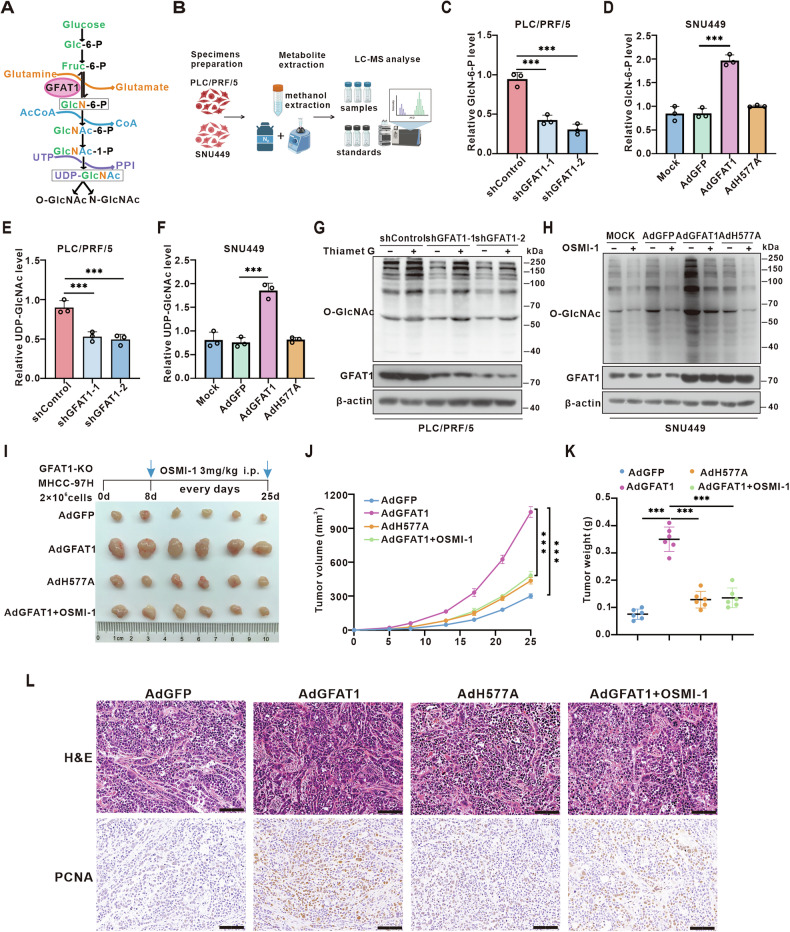
GFAT1 enhances UDP-GlcNAc biosynthesis and protein *O-*GlcNAcylation. **A** Schematic representation of the HBP. Glucose intake feed into the HBP that produces UDP-GlcNAc. GFAT1 was the first rate-limiting enzyme. **B** Extraction and detection of metabolites. **C-F** Relative N-acetylglucosamine-6-phosphate (GlcN-6-P) and uridine diphosphate N-acetylglucosamine (UDP-GlcNAc) levels were measured by LC-MS in GFAT1 knock-down **(C,E)** or overexpression **(D,F)** cells. **G, H** *O-*GlcNAcylation levels of GFAT1 knock-down cells treated with 50 μM Thiamet G (OGA inhibitor) for 12 h before harvesting **(G)** and GFAT1 overexpression cells treated with 25 μM OSMI-1 (OGT inhibitor) for 12 h before harvesting **(H)**. **I–K** Reduced levels of glycosylation inhibited the formation of xenograft tumors in vivo **(I)**. Mice were subcutaneously inoculated GFAT1-KO MHCC-97H cells (AdGFP, AdGFAT1, AdH577A, AdGFAT1+OSMI-1, *n* = 6 per group). Representative images of xenograft tumors were shown and their tumor masses were measured **(J)** and weighed **(K)**. **L** H&E and IHC analysis of PCNA expression in the tumors. Statistical analysis was shown as mean ± SD *(*n = 3). One-way ANOVA followed by the Tukey test, *P < 0.05, **P < 0.01, ***P < 0.001. Scale bar: 100 μm.

Next, we analyzed cellular metabolites in PLC/PRF/5 and SNU449 cells following GFAT1 knockdown or overexpression (Fig. 3A, B). Mass spectrometry analysis of the HBP metabolites revealed a decrease in the relative levels of GlcN-6-P and UDP-GlcNAc (the end products of the HBP) after GFAT1 knockdown (Fig. 3C, E). In contrast, overexpression of GFAT1-WT led to an increase in GlcN-6-P and UDP-GlcNAc levels, whereas the enzyme-inactivating mutant H577A did not show a significant change compared to the control group (Fig. 3D, F). In PLC/PRF/5 cells, global *O-*GlcNAcylation levels were decreased upon GFAT1 knockdown and were restored by the *O*-GlcNAcase (OGA) inhibitor Thiamet G (Fig. 3G). In SNU449 cells, GFAT1-WT overexpression increased *O-*GlcNAcylation levels, whereas the H577A mutant did not induce a noticeable change. Treatment with OSMI-1, a potent inhibitor of *O-*GlcNAc transferase (OGT), led to a significant decrease in *O-*GlcNAcylation levels, suggesting that GFAT1 promotes UDP-GlcNAc biosynthesis and *O-*GlcNAc modification in hepatoma cells in an enzyme activity-dependent manner (Fig. 3H).

Since GFAT1 plays an important role in HCC to activate the HBP flux and increases the *O-*GlcNAcylation levels, we sought to determine whether GFAT1-mediated *O-*GlcNAc signaling contributes to HCC progression. Cell proliferation and colony formation assays demonstrated a reduction of HCC cell proliferation following the addition of OSMI-1 in SNU449 and Huh7 cells (Fig. S3A-D). Furthermore, depletion of *O-*GlcNAcylation markedly inhibited cell migration in these cells (Fig. S3E-H).

To further explore the effect of GFAT1-*O-*GlcNAcylation signal axis on liver cancer cells in vivo, we utilized the CRISPR-Cas9 system to silence endogenous GFAT1 in MHCC-97H cells. Subsequently, GFAT1-WT or GFAT1-H577A were re-expressed, and xenograft transplantation experiments were established in nude mice. Following this, OSMI-1 was administered intraperitoneally in the GFAT1-WT group. Our observations revealed that the enzyme-inactivating mutant H577A did not enhance tumor proliferation. In contrast, GFAT1-WT significantly promoted the formation of transplanted tumors in nude mice, while intraperitoneal administration of OSMI-1 effectively mitigated tumor formation (Fig. 3I-L). Collectively, these data indicate that GFAT1, through its enzymatic function, escalates the HBP flux and UDP-GlcNAc levels. Consequently, this leads to an increase in *O-*GlcNAcylation within liver cancer cells, thereby potentially driving the progression of HCC.

### GFAT1 enhances the *O-*GlcNAcylation of VEZF1

**Fig. 4:**
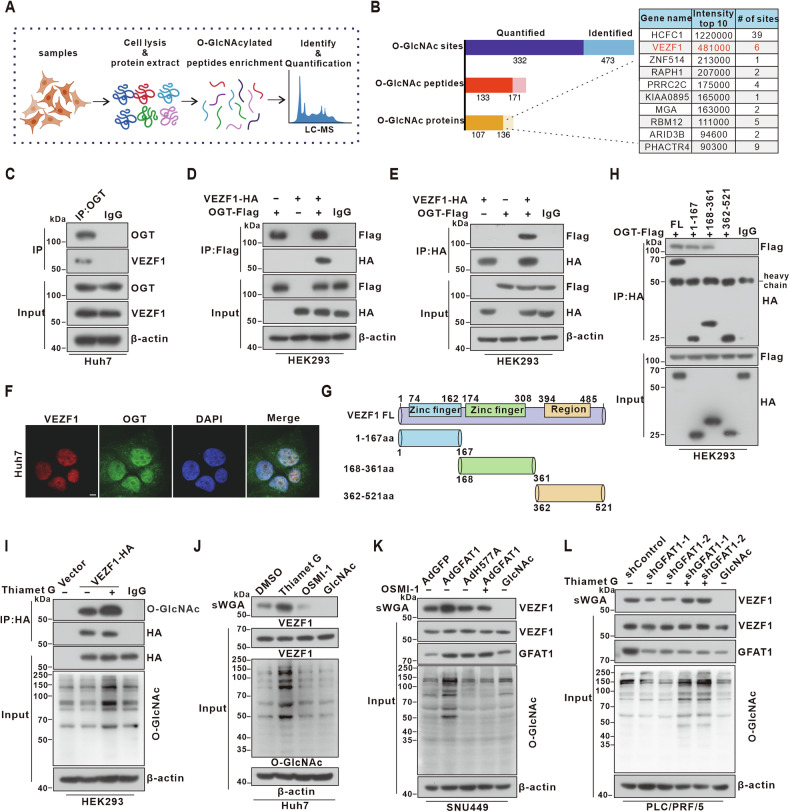
GFAT1 strengthens VEZF1 *O-*GlcNAcylation in hepatoma cells. **A** Flowchart of the enrichment and identification of *O-*GlcNAcylated peptides by proteomic analysis. **B** Summary of the *O-*GlcNAcylated peptides that were quantified and identified. Putative *O-*GlcNAc-modified proteins were obtained from the top ten rankings of all identified *O-*GlcNAcylated peptides. **C** Co-IP of endogenous OGT and VEZF1 in Huh7 cells. (**D**,**E**) Immunoblot analyses of whole cell lysate (WCL) and Co-IP from HEK293 cells transfected with vectors encoding Flag-tagged OGT (Flag-OGT) and HA-tagged VEZF1 (HA-VEZF1). (**F**) Subcellular colocalization of VEZF1 and OGT in Huh7 cells. The nuclei were stained with DAPI. Scale bar: 5 μm. **G-H** The interaction between Flag-OGT and HA-tagged full-length or truncated VEZF1 (1-167aa, 168-361aa, or 362-521aa), as indicated in the diagram **(G)**, were determined in HEK293 cells by Co-IP **(H)**. **I** Enhancement of *O-*GlcNAc modification of VEZF1 following Thiamet G treatment. HEK293 cells were transfected with HA-VEZF1 or vector control for 48 h and treated with 50 μM Thiamet G for 12 h before harvesting. **J** Regulation of VEZF1 *O-*GlcNAc modification by Thiamet G or OSMI-1. Huh7 cells were treated with 50 μM Thiamet G or 25 μM OSMI-1 for 12 h before harvesting, followed by succinylated wheat-germ agglutinin (sWGA) pull-down assay. The monosaccharide inhibitor GlcNAc (20 mM) was added as a negative control during the affinity purification of sWGA-lectin. **K-L** Detection of *O-*GlcNAc modification levels of VEZF1 by sWGA pull-down assay in GFAT1 overexpressing cell treated with 25 μM OSMI-1 **(K)** or in GFAT1 knockdown cell treated with 50 μM Thiamet G **(L)**.

Given the pivotal role of *O-*GlcNAcylation in promotion of HCC progression driven by high GFAT1 expression, 4D-label-free quantitative *O-*GlcNAcylomic analysis was performed to investigate its underlying mechanisms (Fig. 4A). This analysis identified and quantified 473 unique *O-*GlcNAcylated sites, 171 *O-*GlcNAcylated peptides, and 136 candidate *O-*GlcNAcylated proteins in GFAT1 overexpressing group. Notably, most of these *O-*GlcNAcylated proteins were predominantly located in the nucleus (Fig. S4A). Following a systematic ranking of *O-*GlcNAcylation intensities and excluding HCFC1, which has been previously characterized [22], vascular endothelial zinc finger 1 (VEZF1) emerged as a protein of interest (Fig. 4B). As a transcription factor belonging to the zinc finger protein family, VEZF1 is recognized for its substantial impact on tumor progression [19]. Moreover, affinity chromatography using succinylated wheat germ agglutinin (sWGA) beads, which selectively bind to *O-*GlcNAc, revealed a pronounced increase in *O-*GlcNAcylation levels of VEZF1 in human HCC tissue samples with relatively high GFAT1 expression (Fig. S4B).

OGT (*O-*GlcNAc transferase) is the sole enzyme responsible for the *O-*GlcNAcylation of multiple proteins. Endogenous interactions between OGT and VEZF1 were revealed using co-immunoprecipitation (Co-IP) experiments in Huh7 cells (Fig. 4C). Meanwhile, we co-expressed Flag-OGT and HA-VEZF1 expressing plasmids in HEK293 cells. Exogenous Co-IP experiments confirmed the interaction between OGT and VEZF1 (Fig. 4D, E). Moreover, the colocalization of VEZF1 and OGT mostly occurred in the nucleus, as suggested by the immunofluorescence data (Fig. 4F). To map the interaction domain of VEZF1 and OGT, we constructed serial truncated mutants of HA-tagged VEZF1. Co-IP results indicated the binding of OGT to the 1-167aa and 168-361aa regions of VEZF1, both of which contain zinc finger domains (Fig. 4G, H).

Then, we investigated the *O-*GlcNAc modification of VEZF1 in hepatoma cells. Immunoprecipitation of HA-tagged VEZF1 showed a significant enhancement of *O-*GlcNAc modification signal upon treatment with the OGA inhibitor Thiamet G (Fig. 4I). Furthermore, the sWGA assays also confirmed the *O-*GlcNAcylation of endogenous VEZF1 (Fig. 4J). Additionally, we found that overexpression of GFAT1 promoted VEZF1 *O*-GlcNAcylation, while its knockdown had the opposite effect (Fig. 4K, L). Collectively, these findings demonstrate that GFAT1 increases the *O*-GlcNAc modification level of VEZF1 in HCC cells.

### *O*-GlcNAcylation at Ser123 and Ser124 stabilizes VEZF1 by impeding its ubiquitination

**Fig. 5:**
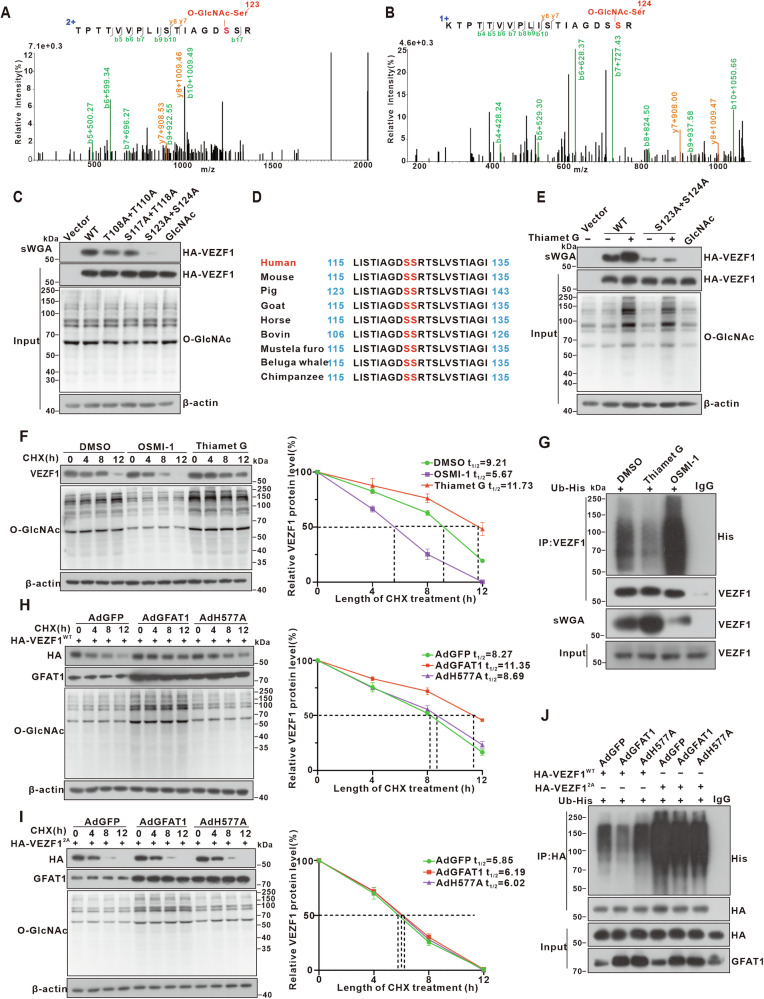
*O-*GlcNAcylation stabilizes VEZF1 by suppressing its ubiquitination. **A,B** Identification of the *O-*GlcNAc modified sites, Ser123 and Ser124, on VEZF1 by mass spectrometry. **C** HEK293 cells were transfected with vector control, HA-WT-VEZF1, or mutants as indicated for 48 h, followed by sWGA pull-down. **D** Cross-species protein sequence alignment of VEZF1. **E** The sWGA pull-down assay in Huh7 cells expressing HA-WT-VEZF1 or Ser123A and Ser124A site-associated mutant. Cells were treated with 25 μM Thiamet G for 12 h before harvesting. **F** The half-life of endogenous VEZF1 in Huh7 cells by immunoblotting and quantitative analysis. Cells were treated with DMSO, 25 μM OSMI-1, or 50 μM Thiamet G for 12 h before harvesting, and protein synthesis was blocked by treatment with 200 μM cycloheximide (CHX) for the indicated times. VEZF1 levels were normalized to those of β-actin, and the 0 h points were arbitrarily set to 100%. Data are representative of at least three independent experiments. (*n* = 3, performed in triplicate) **G** Ubiquitination of endogenous VEZF1 protein in Huh7 cells detected by immunoprecipitation with anti-VEZF1 antibodies. Cells were transfected with His-ubiquitin for 48 h and treated with DMSO, 25 μM OSMI-1, or 50 μM Thiamet G for 12 h before harvesting. **H-I** The half-life of HA-tagged VEZF1^WT^ **(H)** or VEZF1^2A^ mutant **(I)** in Huh7 cells overexpressing GFAT1-WT or H577A. (*n* = 3, performed in triplicate) **J** Ubiquitination of HA-tagged VEZF1^WT^ or VEZF1^2A^ mutant in Huh7 cells overexpressing GFAT1-WT or H577A.

Through 4D-label-free quantitative *O*-GlcNAcylomic analysis, we identified six potential *O*-GlcNAcylation sites on VEZF1, including threonine-108 (T108), threonine-110 (T110), serine-117(S117), threonine-118 (T118), serine-123 (S123), and serine-124 (S124) (Fig. 5A, B). To validate these sites in HCC cells, we generate VEZF1 double mutants by substituting the identified residues with alanine. Our results revealed that S123A and S124A double mutants (VEZF1^2A^) markedly suppressed *O*-GlcNAcylation levels of VEZF1 compared to the VEZF1^WT^ (Fig. 5C). Notably, residues S123 and S124 are highly conserved among different species (Fig. 5D). Furthermore, treatment with Thiamet G, which elevates overall cellular *O*-GlcNAcylation levels, resulted in enhanced *O*-GlcNAcylation in VEZF1^WT^, indicating a response to OGA inhibition. In contrast, the *O*-GlcNAcylation levels of VEZF1^2A^ remained unaltered following Thiamet G treatment (Fig. 5E).

*O*-GlcNAc modification is known to regulate various aspects of protein function, including protein-protein interaction, stability, subcellular localization, and enzyme activity. Immunofluorescence analysis revealed that Thiamet G treatment increased VEZF1 protein levels, while OSMI-1 had the opposite effect, with no significant nuclear translocation observed (Fig. S5A). Our results showed that the protein levels of VEZF1 was decreased upon treatment with the OGT inhibitor OSMI-1, while being augmented by OGA inhibitor Thiamet G (Fig. 5F). The primary protein degradation pathways in eukaryotic cells involve the ubiquitin-proteasome and lysosome pathways. We found that the proteasome inhibitor MG132 significantly reversed the downregulation of VEZF1 induced by OSMI-1 treatment (Fig. S5B). These observations indicated that *O*-GlcNAcylation may regulate the protein stability of VEZF1 through the ubiquitin-proteasome pathway. Consistent with this hypothesis, Thiamet G treatment decreased the ubiquitination level of VEZF1, whereas OSMI-1 increased its ubiquitination (Fig. 5G). In conclusion, the above results indicate that *O*-GlcNAc modification inhibits VEZF1 ubiquitination and thereby enhances its protein stability.

Further investigation revealed that overexpression of GFAT1-WT increased the protein levels of VEZF1 without altering its mRNA levels (Fig. S5C, D). Moreover, compared to VEZF1^WT^, the VEZF1^2A^ mutant exhibited a shortened half-life and increased ubiquitination levels (Fig. 5H–J). These data showed that elevated GFAT1 enhances the protein stability of VEZF1 by increasing its *O*-GlcNAcylation levels, which in turn inhibits its ubiquitination and subsequent proteasomal degradation.

### VEZF1 *O*-GlcNAcylation promotes HCC proliferation and invasion by activating *TNS1* transcription

**Fig. 6:**
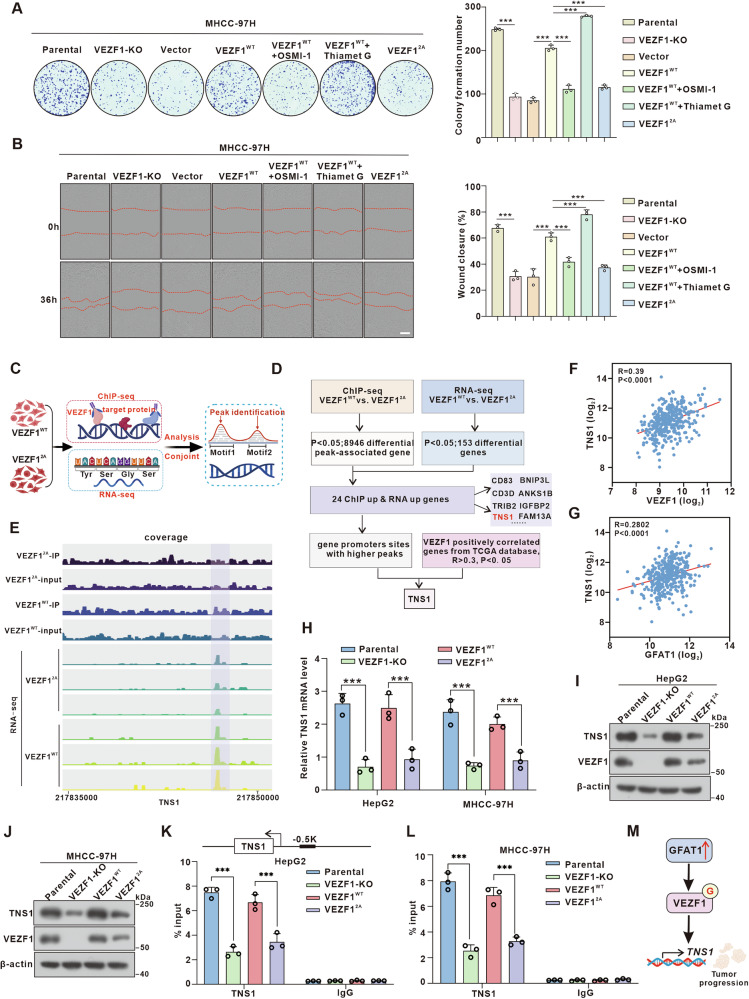
VEZF1 *O-*GlcNAcylation promotes the transcription of TNS1 in HCC. MHCC-97H cells were transfected with the lentiCRISPR-V2 vectors obtained sgRNA targeting VEZF1 to silence endogenous VEZF1. VEZF1-KO cells were stably subcultured and selected in the medium containing 2 μg/mL puromycin, and subsequently infected with adenoviruses expressing VEZF1 (WT or 2 A). **A** Colony formation capacity and quantified results of MHCC-97H cells (*n* = 3 independent experiments). **B** Representative and quantified results of the wound healing assays in MHCC-97H cells. **C** ChIP-seq and RNA-seq were used to identify the downstream genes regulated by VEZF1 *O-*GlcNAcylation. **D** Bioinformatics analysis filtered *TNS1* as a downstream target gene. **E** Data visualization of TNS1 in the genome. **F** Gene expression correlation between *TNS1* and *VEZF1* (r = 0.39, P < 0.0001) from the TCGA database. **G** Gene expression correlation between *TNS1* and *GFAT1* (r = 0.2802, P < 0.0001) from the TCGA database. **H** qPCR analysis of *TNS1* expression in HepG2 and MHCC-97H (*n* = 3). **I-J** Western blotting was used to detect the protein expression levels of VEZF1 and TNS1. **K-L** The transcriptional regulation of *TNS1* gene by VEZF1 was detected by ChIP-qPCR. Cells are treated as directed. IgG was the negative control. **M** Model depicting the mechanism that GFAT1 upregulates TNS1 expression by promoting VEZF1 *O-*GlcNAcylation. Data are shown as the mean ± SD (*n* = 3). Statistical analysis was performed using one-way ANOVA with Tukey’s test. *P < 0.05, **P < 0.01, ***P < 0.001.

To elucidate the role of VEZF1 *O*-GlcNAcylation in HCC progression, we used the CRISPR-Cas9 system to silence endogenous VEZF1 in hepatoma cells. Subsequently, the adenovirus system was used to re-express VEZF1^WT^ or VEZF1^2A^, and after re-expressing VEZF1^WT^, the cells were treated with OGT inhibitor OSMI-1 and OGA inhibitor Thiamet G respectively. A significant reduction in the migratory and proliferative abilities of VEZF1-KO MHCC-97H cells, which were substantially restored by re-expression of VEZF1^WT^, but not VEZF1^2A^. Importantly, we also found that OSMI-1 reduced the proliferation and migration capacity of VEZF1^WT^ overexpressed cells, the opposite results were obtained with TMG treatment, (Fig. 6A, B and Fig. S6A, B), indicating the crucial role of VEZF1 *O*-GlcNAcylation in HCC progression.

Next, to explore the mechanism underlying the role of the transcription factor VEZF1 *O*-GlcNAcylation in HCC progression, we silenced endogenous VEZF1 in HepG2 cells using lentivirus-encoded sgRNA and then re-expressed VEZF1^WT^ or VEZF1^2A^. ChIP-seq and RNA-seq were performed (Fig. 6C). ChIP-Seq data identified 8946 differential peak-associated genes, 1.53% of which were in the promoter region (Fig. 6D and Fig. S7A). Gene ontology (GO) analysis revealed an association of many of these genes with cell adhesion, implicating VEZF1 *O*-GlcNAcylation in tumor development and metastasis (Fig. S7B). Moreover, RNA-seq results identified 153 differentially expressed genes between the two groups (Fig. 6D). Integrating ChIP-Seq and RNA-seq data, we identified 24 target genes with upregulated expression in the VEZF1^WT^ group (Fig. S7C). Among these candidates, *TNS1*, a member of the cell adhesion protein family, showed a high positive correlation with *VEZF1* and *GFAT1* mRNA levels in TCGA-LIHC database (Fig. 6F-G) and is known to act as an oncogene in various tumors [23–26]. Recent studies have demonstrated that TNS1 knockdown reduces HCC cell proliferation, YAP activation, and the formation of cell invasive structures, highlighting its role in HCC development [27]. Therefore, we focus on TNS1 to elucidate the mechanism by which VEZF1 *O*-GlcNAcylation promotes HCC, particularly under conditions of high GFAT1 expression.

Notably, TNS1 expression was reduced at both the mRNA (Fig. 6H) and protein levels (Fig. 6I, J) in VEZF1-KO cells. However, re-expression of VEZF1^WT^, but not VEZF1^2A^, led to increased TNS1 expression at both levels (Fig. 6H, I, J). ChIP-qPCR assays confirmed that VEZF1 transcriptionally activates *TNS1*, an effect diminished by VEZF1^2A^ in HepG2 and MHCC-97H cells, supporting that VEZF1 *O*-GlcNAcylation promotes *TNS1* expression (Fig. 6K, L). The dual-luciferase reporter assay showed reduced binding affinity at the promoter region of *TNS1* following mutation of VEZF1 glycosylation site (Fig. S7D). Subsequently, in the MHCC-97H liver cancer cell line with endogenous VEZF1 knockout, silencing *TNS1* gene inhibited the promoting effect of VEZF1^WT^ on HCC progression. Conversely, overexpression TNS1 was able to reverse the inhibitory effect of VEZF1^2A^ on HCC progression (Fig. S7E-I). Furthermore, we observed the regulation of TNS1 expression in the presence of GFAT1. The results indicated that high expression of GFAT1 could enhance the VEZF1 *O*-GlcNAcylation, thereby increasing both mRNA and protein levels of TNS1. Compared to the mutant, re-expression of VEZF1^WT^ more effectively facilitated the TNS1 expression (Fig. S8A-C). Collectively, these findings suggest that elevated GFAT1 expression triggers the activation of the VEZF1-TNS1 axis, thereby promoting HCC progression (Fig. 6M).

### *O*-GlcNAcylation of VEZF1 is crucial for HCC progression in vivo

**Fig. 7:**
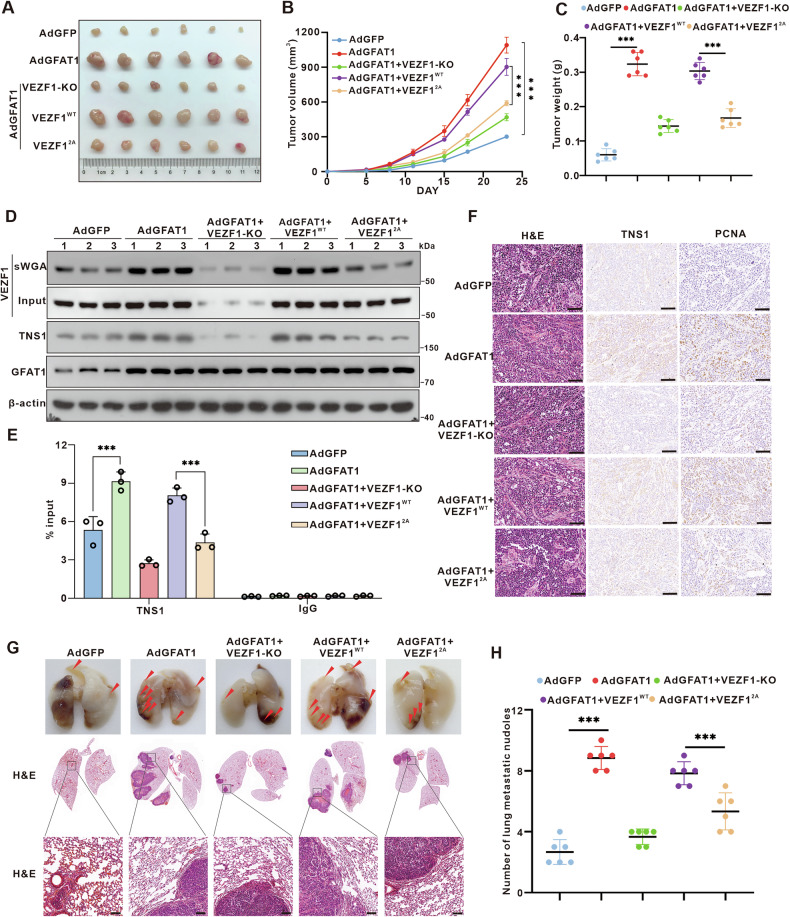
GFAT1-mediated VEZF1 *O-*GlcNAcylation promotes oncogenesis and metastasis of HCC in vivo. **A–C** GFAT1-regulated VEZF1 *O-*GlcNAcylation promote xenograft tumor formation in vivo. AdGFP, AdGFAT1, AdGFAT1 + VEZF1 KO, AdGFAT1 + VEZF1^WT^, or AdGFAT1 + VEZF1^2A^ MHCC-97H cells were injected subcutaneously into the axillae of nude mice (n = 6 for each group). Mice were euthanized after 21 days, and their tumor masses were excised **(A)**, measured and weighed **(B, C)**. **D** sWGA and Western blot were used to detect the *O-*GlcNAcylation of VEZF1 and the protein expression of TNS1. **E** ChIP-qPCR analysis showed that VEZF1 *O-*GlcNAcylation promoted *TNS1* transcriptional expression in vivo. **F** IHC analysis of GFAT1, TNS1 and PCNA in the xenograft tumor samples. Scale bar, 100 μm. **G** Gross appearances of lungs with tumors and H&E staining of sections of metastasized lungs. Scale bar, 100 μm. **H** The number of tumor metastatic nodules. Data are represented as mean ± SD, and analyzed by one-way ANOVA followed by Tukey’s test, *P < 0.05, **P < 0.01, ***P < 0.001.

To further ascertain the role of GFAT1-regulated VEZF1 *O*-GlcNAcylation in tumor growth and metastasis in vivo, we performed xenograft tumor experiments in nude mice (Fig. 7A). The subcutaneous xenograft tumor model showed that elevated GFAT1 expression significantly accelerated tumor growth rate compared to the control group. Mice injected with VEZF1^WT^ cells showed more rapid tumor growth and greater tumor weight than those injected with VEZF1^2A^ mutant (Fig. 7B, C). The sWGA results further confirmed that GFAT1 enhanced the *O-*GlcNAcylation of VEZF1, and the glycosylation level of VEZF1 was markedly reduced by the mutation of the *O*-GlcNAcylation sites. Moreover, immunoblotting results showed that the expression levels of TNS1 were consistent with the *O*-GlcNAc modification levels of VEZF1 (Fig. 7D). ChIP-qPCR assays further substantiated that VEZF1 *O-*GlcNAcylation stimulates *TNS1* transcriptional expression (Fig. 7E). Subsequent IHC analysis revealed that the expression of TNS1 and proliferating cell nuclear antigen (PCNA) was upregulated in the GFAT1-overexpressing group compared with the control group. Additionally, there was a significant increase in TNS1 expression and PCNA accumulation in tissues from the VEZF1^WT^-injection group compared to the VEZF1^2A^ mutant-injection group (Fig. 7F). These results underscore the pivotal role of VEZF1 *O*-GlcNAcylation in promoting liver tumor growth in vivo.

Subsequently, we established a pulmonary metastasis model by tail vein injection of hepatoma cells to evaluate the impact of VEZF1 *O*-GlcNAcylation on HCC metastasis in vivo. After 5 weeks, mice were euthanized, and the lung tissues were collected. Hematoxylin and eosin (H&E) staining of the lungs confirmed that their pulmonary metastases formed solid tumors (Fig. 7G, H). Meanwhile, overexpression of GFAT1 increased the number of pulmonary metastatic foci, and re-expression of VEZF1^WT^ in VEZF1-KO cells led to a significant increase in lung metastasis compared to VEZF1^2A^ mutant. Together, these findings clearly establish VEZF1 *O*-GlcNAcylation as a crucial factor in HCC progression and pulmonary metastasis in mouse models.

### Therapeutic targeting the GFAT1-VEZF1 axis suppresses HCC progression

**Fig. 8:**
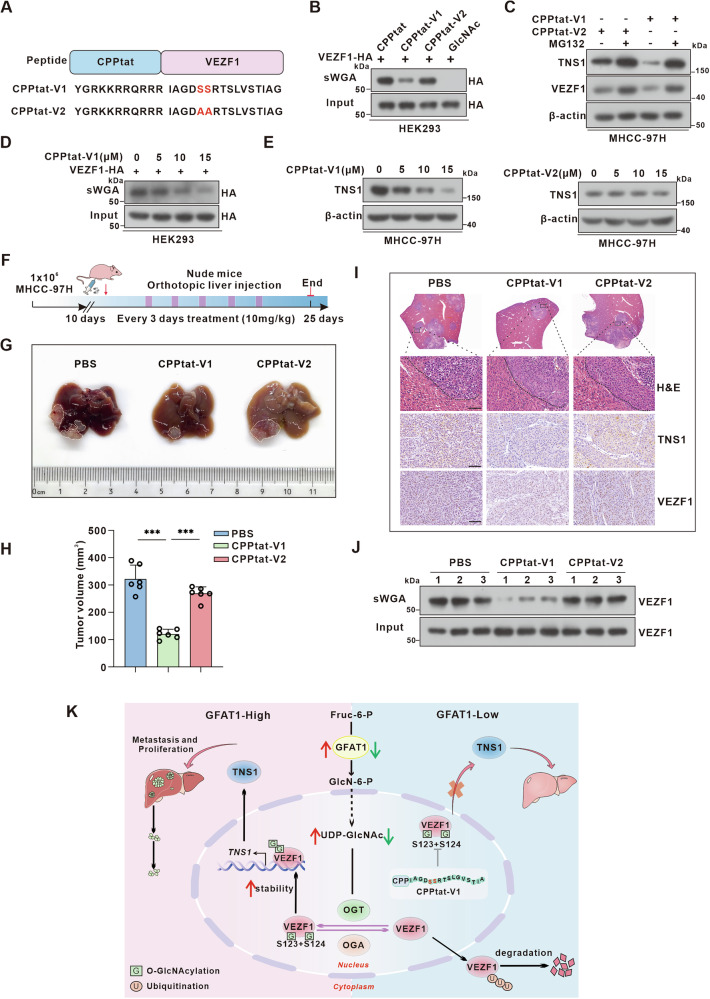
Therapeutic inhibitions of the glycosylation modification of VEZF1 suppress liver cancer progression. **A** Schematic of CPPtat-V1 and CPPtat-V2 peptides. The different residues are shown in red. **B** HEK293 cells transfected with VEZF1 plasmid were treated with 10 μM CPPtat, CPPTAT-V1 and CPPTAT-V2 for 24 h, and the glycosylation modification level of VEZF1 was detected by sWGA. **C** Western blot analysis for the expression of VEZF1 and TNS1 in MHCC-97H cells treated with CPPtat-V1 and CPPtat-V2 with or without MG132. **D** sWGA analysis for the glycosylation modification level of VEZF1 in cells treated with CPPtat-V1 for 0, 5, 10, 15 μM. **E** Western blot analysis for the expression of TNS1 in MHCC-97H cells treated with CPPtat-V1 or CPPtat-V2 for 0, 5, 10, 15 μM. **F** Schematic diagram for timeline of CPPtat, CPPtat-V1 and CPPtat-V2 treatment in the nude mice. **G** Representative photos of mice treated with CPPtat, CPPtat-V1 and CPPtat-V2. **H** tumor weights were measured. n = 6 per group, two-tailed unpaired t test. **I** Representative images of H&E staining, IHC analysis of TNS1 and VEZF1 in the tumor samples. Scale bar, 80 μm. **J** sWGA were used to detect the *O-*GlcNAcylation of VEZF1. **K** Schematic representation of the GFAT1-VEZF1-TNS1 signaling axis in HCC progression.

Our finding that GFAT1-mediated *O*-GlcNAcylation of VEZF1-Ser123 and VEZF1-Ser124 plays a pivotal role in transcriptional activation of *TNS1*, suggesting that therapeutic potential for HCC may be conferred by targeting the GFAT1-VEZF1-TNS1 signaling axis through inhibition of this specific modification. Based on the identified glycosylation modified peptide of VEZF1 (119-134), sequence containing S123 and S124 (V1) or the S123A and S124A mutations (V2), we designed cell-penetrating peptides (CPPs) to introduce a competitive inhibitor of VEZF1 *O*-GlcNAcylation into HCC cells. A CPP was fused to the V1 (CPPtat-V1) and V2 (CPPtat-V2) sequences as shown in Fig. 8A. Importantly, we found that CPPtat-V1, but not CPPtat-V2, effectively reduced *O*-GlcNAcylation of VEZF1 (Fig. 8B). Consistent with these findings, the CPPtat-V1 peptide, but not the CPPtat-V2 peptide, significantly reduced endogenous TNS1 expression, an effect that was reversed by the proteasome inhibitor MG132 (Fig. 8C). Furthermore, the level of VEZF1 *O*-GlcNAcylation decreased with increasing concentrations of CPPtat-V1 (Fig. 8D). In addition, the CPPtat-V1 peptide decreased TNS1 expression in a dose-dependent manner, whereas no obvious change was observed in cells treated the control CPPtat-V2 peptide (Fig. 8E). Collectively, these results indicate that CPPtat-V1 inhibits *O*-GlcNAc modification of VEZF1 and induces its proteasomal dependent degradation, thereby reducing TNS1 expression in HCC cells.

Importantly, CPPtat-V1 treatment significantly impeded HCC proliferation and migration (Fig. S9A–D). To further assess the in vivo efficacy of the CPPtat-V1 peptide, MHCC-97H cells were injected orthotopically into the liver of nude mice. Ten days after tumor colonization, mice were administered 10 mg/kg of control CPPtat or CPPtat-V1 intraperitoneally every other day (Fig. 8F). Consistent with our in vitro results, CPPtat-V1 treatment significantly suppressed HCC progression and reduced the area of liver lesions in mice (Fig. 8G, H). Subsequent IHC analysis revealed decreased expression of TNS1 and VEZF1 in the CPPtat-V1 group compared to the PBS and CPPtat-V2 groups (Fig. 8I). Additionally, the sWGA results further confirmed that CCPtat-V1 restrained the *O*-GlcNAcylation of VEZF1 (Fig. 8J). Overall, the findings demonstrate that CPPtat-V1 inhibits the glycosylation modification of VEZF1, thereby suppressing HCC progression.

## Discussion

Metabolic reprogramming has been identified as a pivotal hallmark that elucidates the complexities of tumorigenesis [3, 28]. The HBP, a critical branch of the glycolytic pathway, is instrumental in the conversion of glucose and other metabolites into uridine diphosphate N-acetylglucosamine (UDP-GlcNAc), a precursor for protein *O-*linked N-acetylglucosamine (*O-*GlcNAc) modifications [29, 30]. The flux through the HBP and the ensuing *O*-GlcNAcylation of proteins are tightly regulated processes that can significantly influence cellular metabolic pathways and signal transduction, with aberrations in this modification closely associated with diverse cancer characteristics [31]. As the first rate-limiting enzyme of the HBP, GFAT1’s expression is intimately connected to glycosylation in cancer cells [14, 32, 33]. Our study provides compelling evidence that GFAT1 is not only highly expressed in HCC but also functions to enhance HBP flux and *O*-GlcNAcylation of the transcription factor VEZF1, thereby promoting the proliferation and migration of liver cancer cells (Fig. 8K).

VEZF1 is a well-characterized transcription factor involved in the pathogenesis of various cancers [18, 19, 34, 35]. Its N-terminus contains six Cys2/His2-type zinc finger motifs known to promote DNA binding, and its C-terminus contains glutamine extensions and proline-rich regions that can bind to the CT/GC-rich region in *IL-3* promoters, modulating transcriptional activation or repression. Meanwhile, VEZF1 also binds to and activates the *ET-1* promoter in human vascular endothelial cells, thereby regulating angiogenesis [17]. Overexpression of VEZF1 has been shown to increase osteosarcoma cell proliferation, invasion, and migration, while decreasing apoptosis [35]. Additionally, VEZF1 activates SETBP1 transcription, and the SETBP1/SET/PP2A oncogenic signaling axis promotes the malignant phenotype of ovarian cancer cells [18]. In HCC, VEZF1 transcriptionally activates PAQR4 to accelerate HCC progression [19].

Post-translational modifications (PTMs) of VEZF1 are particularly important in cancer research. For example, the E3 ubiquitin ligase STUB1 mediated ubiquitination can reduce the stability of VEZF1 and attenuate liver cancer progression [19]. Although the roles of VEZF1 acetylation and phosphorylation are less characterized, PTM databases such as PhosphoSitePlus (www.phosphosite.org) suggest potential acetylation sites at K100 and K362, and phosphorylation sites at Ser197, Ser256 and Ser518. The precise biological significance of these modifications remains to be elucidated. Crosstalk between protein PTMs plays a crucial role in the occurrence and development of tumors [36, 37]. Our research indicates that GFAT1-mediated *O*-GlcNAcylation can enhance the stability of VEZF1 by inhibiting its proteasomal degradation, thereby promoting the progression of HCC. Loss of *O*-GlcNAcylation by the VEZF1^2A^ (S123A + S124A) mutation shortened VEZF1 half-life by enhancing its ubiquitination and degradation, thus dampening the carcinogenic role of GFAT1. Future studies should further investigate the cross-talk between VEZF1 *O*-GlcNAcylation and other PTMs in HCC progression. In addition, our design of a potentially bioactive CPPs based on VEZF1 glycosylation site, which competitively inhibits VEZF1 *O*-GlcNAcylation, shows therapeutic potential in vitro and in vivo, suggesting a future therapeutic application for liver cancer.

Tensins, consisting of four members in mammals (TNS1, TNS2, TNS3 and TNS4), are a family of cellular-adhesion constituents that have been extensively studied [38]. In intrahepatic cholangiocarcinoma (iCCA), SOX17 has been identified as a transcriptional regulator of TNS4, thereby facilitating tumor growth [39]. Concurrently, deletion of MLL3 reduces levels of H3K4me1 and H3K27ac at the *TNS3* enhancer, consequently inhibiting TNS3 expression [40]. Through the integration of ChIP-Seq and RNA-Seq analyses, we have discovered that VEZF1 can enhance *TNS1* transcription. TNS1 plays an important role in cell biology, especially in cell adhesion, polarization, migration, invasion, proliferation, apoptosis, and mechanical transduction [41]. Abnormal expression of TNS1 in vivo is associated with various diseases, especially tumors. In gastric cancer, TNS1 promotes proliferation and metastasis by enhancing cell contraction [25, 42]. TNS1 also promotes the progression of colorectal cancer and represents a potential therapeutic target [43, 44]. Importantly, TNS1 functions in mechanotransduction and activates YAP through its interaction with integrin β1 to promote HCC progression induced by advanced glycation end-products (AGEs). Notably, knockdown of TNS1 resulted in a significant reduction in the number of transformed foci within HCC cells stimulated by AGEs, thereby further substantiating the critical role of TNS1 in tumorigenesis [27]. Our findings indicate that VEZF1-WT, in contrast to VEZF1-2A, enhances the transcription of TNS1, with a positive correlation between the expressions of VEZF1 and TNS1 in HCC, as well as between TNS1 and GFAT1. These results imply that GFAT1-regulated *O*-GlcNAcylation of VEZF1 facilitates TNS1 transcription.

Our study has some limitations that warrant future investigation. The impact of *O-*GlcNAcylation on protein structure of VEZF1 warrants further investigation. VEZF1 is crucial for vascular development and serves as a significant target in drug research through its interactions with various molecules [45, 46], and the impact of *O-*GlcNAc modification on these interactions requires additional study. Although the glycosylation modification of VEZF1 was found to play an important role in HCC, we can not exclude the contribution of other *O*-GlcNAcylation proteins regulated by GFAT1. Additionally, to gain a complete understanding of the biological roles of GFAT1 and VEZF1 in HCC, future studies utilizing mouse models specifically deficient in GFAT1 or VEZF1 are necessary.

In conclusion, GFAT1 is abnormally overexpressed in HCC and associated with poor prognosis in patients. Its upregulation increases HBP flux and VEZF1 *O*-GlcNAcylation level, which in turn promotes the malignant progression of liver cancer. In addition, VEZF1 *O*-GlcNAcylation of Ser123 and Ser124 inhibits its ubiquitination and degradation, enhances protein stability, and improves the ability of transcriptional regulation, leading to abnormal accumulation of oncogenic product TNS1, thus supporting the malignant phenotype of cancer cells. These findings also highlight the therapeutic potential of targeting the GFAT1-VEZF1-TNS1 signaling axis in HCC.

## Materials and methods

Full details are available in Supplementary Material and Methods.

### Clinical specimens

HCC tumor tissues and paired non-tumorous tissue samples were collected from 42 patients undergoing surgery at the second affiliated hospital of Chongqing Medical University. All patients provided an informed consent and had not received chemotherapy or radiation therapy before surgery. This study was approved by Institutional Ethical Review Board of Chongqing Medical University (reference number: 2024062).

### Adenovirus production

The amplified GFAT1-WT, the mutants of GFAT1 (H577A), VEZF1^WT^, VEZF1^2A^ fragment was inserted into the shuttle vector pAdTrack-TO4 (kindly provided by Dr. Tong-Chuan He, University of Chicago, USA). And then, recombinant adenoviruses, AdGFAT1, AdH577A, AdVEZF1^WT^ and AdVEZF1^2A^ were produced using the AdEasy system as previously described [33]. AdGFP was used as a negative control.

### Co-immunoprecipitation (Co-IP)

For the interactions of exogenous proteins, cells were co-overexpressed with indicated plasmids and then harvested after 48 h. The cells were lysed by Cell lysis buffer for Western and IP (P0013, Beyotime, Shanghai, China) containing 1× Protease Inhibitor (C0001, TargetMol, Wellesley Hills, MA, USA) and 1× Phosphatase Inhibitor (C0003, TargetMol, Shanghai, China). After Ultrasonic cracking and centrifugation, the supernatant liquids were incubated with anti-FLAG, anti-HA, or control IgG antibodies overnight at 4 °C. For the interactions of endogenous proteins, the supernatant liquids were incubated with anti-VEZF1 and anti-*O-*GlcNAc. And subsequently incubated with protein A/G agarose beads (MCE, NJ, USA) for 4 h. Immunoprecipitates were washed, and detected the corresponding protein with the special antibody by Western blotting.

### Animal models

Subcutaneous xenograft model was established in nude mice (NU/NU Mice, the Beijing Charles River Experimental Animal Technology Co., Ltd., Beijing, China). Briefly, 2 × 10^6^ cells were subcutaneously grown in mice (*n* = 6 per group). After tumor formation, the tumor size was observed and recorded every 5 days. Then, tumors were removed, photographed for documentation, and embedded in paraffin for IHC assays.

For the tail vein injection method for lung metastasis model, six-week-old male NU/NU nude mice were randomly divided into five groups (*n* = 6 per group). 2 × 10^6^ MHCC-97H cells were resuspended in 100 μL PBS and injected into the tail vein of the mice. Five weeks after injection, the mice were sacrificed. The lung tissues were collected and fixed in paraformaldehyde.

## Supplementary information

Supplementary Information

WB Data

## Data Availability

All study data are included in the article and Supplementary information. Data of *O-*GlcNAcylation 4D-Label free quantitative proteomic have been deposited in Integrated Proteome Resources (IPX0009754000). The accession numbers for ChIP-Seq data (GSE278232) and RNA-Seq data (GSE278334) sets reported in this article is NCBI Gene Expression Omnibus. Source data supporting the findings of this study are provided with this paper. All supporting data not presented here are available from the corresponding author on reasonable request.
