# Supplementary material for: GFAT1 promotes the progression of hepatocellular carcinoma via enhancing the O-GlcNAcylation of VEZF1
Source: Cell Death Dis. 2025 Aug 26;16(1):647. doi: 10.1038/s41419-025-07975-5 (PMC12381164; doi:10.1038/s41419-025-07975-5)
Supplement: Supplementary file 1 — Supplementary Information [file 41419_2025_7975_MOESM1_ESM.pdf]

GFAT1 promotes the progression of hepatocellular carcinoma via enhancing the O-GlcNAcylation of VEZF1

Jia-yao Yang[1, #], Rong Zhang[1, #], Zhi-rong Zhang[1,#], Shan Li[1], De-ao Gong[1], Chen-hao Li[1], Chang Chen[2], Lu-yi Huang[1], Ai-long Huang[1], Ni Tang[1,*], Kai Wang[1,*]

1 Key Laboratory of Molecular Biology for Infectious Diseases (Ministry of Education), Institute for Viral Hepatitis, Department of Infectious Diseases, The Second Affiliated Hospital, Chongqing Medical University, Chongqing 400010, China.

2 College of pharmacy, Chongqing Medical University, Chongqing 400016, China.

* Corresponding author. Ni Tang, E-mail: nitang@cqmu.edu.cn;

* Corresponding author. Kai Wang, E-mail: wangkai@cqmu.edu.cn;
Tel: 86-23-68486780, Fax: 86-23-68486780

# These authors contribute equally to this work.

This PDF file includes:

Materials and methods.

Supplementary Figures and Figure Legends.

Supplementary Table.

Materials and methods

Clinical specimens

HCC tumor tissues and paired non-tumorous tissue samples were collected from 42 patients undergoing surgery at the second affiliated hospital of Chongqing Medical University. All patients provided an informed consent and had not received chemotherapy or radiation therapy before surgery. This study was approved by Institutional Ethical Review Board of Chongqing Medical University (reference number: 2024062).

Public database analysis

Gene expression data and corresponding clinical data for 374 patients with HCC were obtained from The Cancer Genome Atlas Liver Hepatocellular Carcinoma (TCGA-LIHC) dataset (https://www.cancer.gov/ccg/research/genome-sequencing/tcga).The GSE14520 dataset (https://www.ncbi.nlm.nih.gov/geo/query/acc.cgi?acc=GSE14520) was obtained from the GEO database.

Cell cultures and reagents

SNU449, PLC/PRF/5 cells were obtained from the American Type Culture Collection (Manassas, VA, USA), MHCC-97H, Huh7, and HEK293T cells were from the Cell Bank of the Chinese Academy of Sciences (Shanghai, China). Immortalized human hepatocytes (MIHA) were a gift from Dr. Ben C.B. Ko, Hong Kong Polytechnic University, China. All cell lines were assured free of mycoplasma by the GMyc-PCR Mycoplasma Test Kit (Yeasen, Shanghai, China). HepG2, Huh7, PLC/PRF/5, MHCC97-H, MIHA and HEK293T cells were cultured in DMEM medium (Gibco) supplemented with 10% fetal bovine serum (FBS; Natocor, Cordoba, Argentina) and 1% Penicillin-Streptomycin (MedChemExpress, MCE; NJ, USA). SNU-449 was cultured in RMPI-1640 medium (Gibco, NY, USA) supplemented with 10% FBS (Natocor) and 1% Penicillin-Streptomycin (MCE).

Plasmid construction

The full-length cDNA of Human GFPT1, OGT, VEZF1 and the truncated mutants VEZF1-1-167aa, VEZF1-168-361aa, VEZF1-362-521aa were amplified by PCR and

subcloned into the pAdTrack-TO4 or pBu-3HA. The mutants of GFAT1 (H577A) and VEZF1(VEZF1-S123A&S124A) were constructed by site-directed mutagenesis using wild-type (WT) plasmid as the template. Primers are provided in Supplementary Table S1.

Adenovirus production

The amplified GFAT1-WT, the mutants of GFAT1 (H577A), $VEZF1^{WT}$, $VEZF1^{2A}$ fragment was inserted into the shuttle vector pAdTrack-TO4 (kindly provided by Dr. Tong-Chuan He, University of Chicago, USA). And then, recombinant adenoviruses, AdGFAT1, AdH577A, $AdVEZF1^{WT}$ and $AdVEZF1^{2A}$ were produced using the AdEasy system as previously described[1]. AdGFP was used as a negative control (kindly provided by Dr. Tong-Chuan He, University of Chicago, USA).

Lentivirus production

To knock down GFAT1 expressing, three pairs of short hairpin RNA targeting GFAT1 (Table S2) was designed and inserted into the lentiviral vector pLL3.7 (Prof. Bing Sun, the Shanghai Institute of Biochemistry and Cell Biology, Chinese Academy of Sciences, Shanghai, China). A negative control construct (shCon) was also generated. HEK293T cells were co-transfected with the packaging plasmids pVSV-G, pCMV-Δ8.9 and recombined lentiviral vector pLL3.7 at a ratio of 2:3:4 for lentivirus production using Lipo8000 (Beyotime, Shanghai, China). The CRISPR/Cas9 system was used for GFPT1 and VEZF1 knockout. Single guide RNA sequences targeting VEZF1 were designed (Supplementary Table S2) and cloned into the lentiviral vector CRISPR-V2 (Dr. Ding Xue, Tsinghua University, Beijing, China). HEK293T cells were co-transfected with the packaging plasmids VSV-G, gal-pol and recombined lentiviral vector MSCV2.2 at a ratio of 2:2:5 for lentivirus production using Lipo8000 (Beyotime). To overexpression TNS1, the pReceiver-Lv245-TNS1 (EX-A4325-Lv245, Guangzhou iGene Biotechnology Co., Ltd., Guangzhou, China) , pVSV-G and pCMV-Δ8.9 were co-transfected in HEK293T cells. Lentiviruses in the supernatant were collected at 72hrs post transfection.

Cell proliferation assay

A total of 2000 Huh7, PLC/PRF/5, SNU449 or MHCC-97H cells were planted into 96-well plates. Images was taken by IncuCyte HD system (IncuCyte ZOOM, Essen BioScience, USA) every 24h.

Colony formation assay

Cells were planted into 6-well plates (800 cells/well) and cultured for two or three weeks until the colonies were visible. The medium was replaced every two days. Colonies were washed with PBS and fixed with 4 % PFA for 30 min, then the colonies were stained with 0.5 % crystal violet blue for 15 min. Then the plates were washed with ddH_2O and colonies were numbered.

Transwell migration assay

Cell migration was performed using a transwell insert with 8.0 μm pores (Corning). MHCC-97H (3×10^4), Huh7 (4×10^4), PLC/PRF/5 (4×10^4), or SNU449 (3×10^4) cells were seeded onto the upper compartment in serum-free medium. The lower compartment was replete with medium containing 20% FBS (Natocor). The migrated cells were subsequently fixed with 4% paraformaldehyde and stained with crystal violet (Beyotime). For each experiment, the number of migrated cells in five random fields was counted, and three independent experiments were performed.

Wound healing assay

Cells were cultured in 96-well plates until confluent, and wounds were created using WoundMaker™ (Essen Bioscience, MI, USA) on the cell monolayer. The real-time wound areas were recorded by the IncuCyte ZOOM Live-Cell Imaging system (Essen BioScience).

Detection and analysis of UDP-GlcNAc

After washing the cells twice with precooled saline, liquid nitrogen quenching was performed, and subsequently, metabolites were extracted with 400 μL cold methanol. The mixture was centrifuged for 20 min (14,000g, 4 ° C). The supernatant was dried in a vacuum centrifuge. Samples were dissolved in 100 μL of methanol solvent for analysis. Targeted LC-MS/MS

(Agilent 1290 Infinity II–6495C) was used to analyze the level of UDP-GlcNAc. The UDP-GlcNAc standard（HY-112174 , MCE）was prepared for standard curve drawing.

O-GlcNAcylation 4D-Label free quantitative proteomic

The cell samples were sent to Shanghai Applied Protein Technology (Shanghai, China) for mass spectrometry (MS) analysis. Briefly, total protein extracted from the cells was digested into peptides using trypsin. Then the peptides were incubated Anti GlcNAc-S/T antibody beads (PTMScan O-GlcNAc [GlcNAc-S/T] Motif Kit; Cell Signaling Technology) with gentle shaking overnight at 4℃. The beads were washed 3 times with cold IPA buffer and cold water, followed by desalting using a ZipTip. Finally, the samples were separated for LC-MS/MS detection.

Western blotting

Proteins of cells or liver samples were extracted with lysis buffer (P0013, Beyotime Biotechnology, Shanghai, China) containing inhibitors. Protein samples were separated by 10% SDS/PAGE and electro-transferred onto PVDF membranes (IPVH00010, Merck Millipore, Billerica, MA, USA). The membranes were incubated with the indicated primary antibody overnight (Supplementary Table S4), incubated with fluorescence-labeled secondary antibody at room temperature for 1.5 h. Protein expression was visualized with the Enhanced Chemiluminescence system (BioRad, Hercules, CA, USA).

Co-immunoprecipitation (Co-IP)

For the interactions of exogenous proteins, cells were co-overexpressed with indicated plasmids and then harvested after 48 h. The cells were lysed by Cell lysis buffer for Western and IP (P0013, Beyotime) containing 1× Protease Inhibitor (C0001, TargetMol, Wellesley Hills, MA, USA) and 1× Phosphatase Inhibitor (C0003, TargetMol). After Ultrasonic cracking and centrifugation, the supernatant liquids were incubated with anti-FLAG, anti-HA, or control IgG antibodies overnight at 4 °C. For the interactions of endogenous proteins, the supernatant liquids were incubated with anti-VEZF1 and anti-O-GlcNAc. And subsequently incubated with protein A/G agarose beads (MCE) for 4 h. Immunoprecipitates were washed,

and detected the corresponding protein with the special antibody by western blotting.

sWGA pull-down assay

Liver tissues or hepatic cells were lysed in Lysis 125 buffer (50 mM Tris, pH 7.4, 125 mM NaCl, 5 mM EDTA, 5 mM EGTA, 0.1% Nonidet P-40, 50 mM NaF, 1 mM PMSF and 1× Proteinase Inhibitor Cocktail (Beyotime). Before the supernatant was combined with succinylated Wheat Germ agglutinin (sWGA)-conjugated agarose beads (AL-1023S, Vector Laboratories, Burlingame, USA) at 4 °C overnight, PNGase-F (P0704S; New England Biolabs, USA) was used by digesting cells suspension to remove N-linked glycoproteins. Precipitated complexes were washed and subjected to immunoblotting assays with anti-Flag, anti-HA, or anti-VEZF1 antibodies. Before the sWGA binding assay, the target protein of input needed to be adjusted to a similar level[2].

Immunofluorescence (IF)

Cells were fixed with 4% paraformaldehyde for 30 mins, permeabilized with 0.5% Triton X-100 (T8787, Sigma-Aldrich, Germany) for 15 mins, blocked with goat serum (ZLI-9022, ZSGB-BIO, Beijing, China) for 1 h, incubated with the indicated primary antibody overnight. Subsequently, incubated with fluorescence-labeled secondary antibody at 37 ℃ for 2 h, and nuclear staining with 4′,6-Diamidine-2′-phenylindole dihydrochloride (DAPI; Roche Diagnostics GmbH, Mannheim, Germany) for 5 mins. Images were taken using a Leica confocal microscope (Leica TCS SP8, Leica Microsystems, Wetzlar, Germany).

Chromatin immunoprecipitation (ChIP)

Chromatin immunoprecipitationm (ChIP) assay was performed as described[2]. In brief, 6×10^6 cells were cross-linked using 1% paraformaldehyde for 10 min at 37 °C. Cell lysates were sonicated by the Bioruptor at high-output power for 15 cycles (30s ON and 30s OFF). Supernatants were separated and incubated with anti-acetylated VEZF1 (sc-365560, Santa Cruz Biotechnology) or control IgG overnight at 4 °C. Chromatin-antibody complexes were collected by protein A/G agarose beads (MCE), washed and then eluted. DNA complexes were reverse cross-linked in a water bath at 65 °C for 4 h. DNAs were purified with

phenol-chloroform-isopentanol, and ethanol precipitated, and then quantified by real-time PCR. Primers are listed in (Table S3). High through-put sequencing and data analysis were conducted by Seqhealth Technology Co., LTD (Wuhan, China).

Quantitative reverse transcription polymerase chain reaction (qRT-PCR)

TRIzol reagent (Invitrogen, Carlsbad, CA, USA) was used to extract total RNA from tissues or cells. PrimeScript RT Reagent Kit (TaKaRa, Shiga, Japan) was used for cDNA synthesis. The target gene expression was then detected using real-time PCR (SYBR green) with specific primers. The PCR conditions were as follows: 95 °C for 3 min, followed by 40 cycles of 95 °C for 15 s, 60 °C for 15 s, and 72 °C for 20 s, concluding with an extension step of 72 °C for 5 min. The data were analyzed using the 2−ΔΔCt method. The primers used were listed in Supplementary Table S3.

Dual-luciferase reporter assay

TNS1 promoter fragments (-300 to +687) were PCR amplified and cloned into the *Kpn*I and *Bgl*II restriction enzyme sites of pGL3-Basic vector to construct TNS1 firefly luciferase reporters. The TNS1 firefly luciferase reporter plasmid and renilla luciferase reporter control plasmids (pRL-TK) were transfected with Lipo8000 (Beyotime) for 24 hours and then the cells were re-spread. After knocking down VEZF1, adenovirus was used to re-express wild-type and mutant VEZF1. In Dual-Luciferase®Reporter (DLR™) Assay System (Promega, Madison, Wisconsin, USA), the luciferase activity equals to the ratio Fireflfly luciferase/Renilla luciferase.

Animal models

This study was approved by the Institutional Ethical Review Board of Chongqing Medical University. All mouse experiments were approved by the Research Ethics Committee of Chongqing Medical University (Approval No: IACUC-CQMU-2023-10004) and followed all relevant animal welfare guidelines.

For the chemically induced HCC model, two-week-old C57BL/6 mice were given an initial intraperitoneal injection of diethylnitrosamine (DEN; 50 mg/kg) and followed by the

administration of CCl_4 (2 mL/kg, twice a week for 12 weeks), commencing at four weeks of age.

For the SB100/AKT/NRAS induced HCC model, five-week-old male C57BL/6 mice were injected with a plasmid equal to 10% of mouse body weight. For instance, a mouse weighing 15 g received an injection volume of 1.5 mL. Plasmid solutions were prepared as follows: 1 μg of pCMV-CAT-T7-SB100, 6 μg of pT3-myr-AKT-HA, and 6 μg of pT/CAGGS-NrasV12 dissolved in 1.5 mL of saline. The injection was controlled to be completed within 5-7 seconds.

Subcutaneous xenograft model was established in nude mice (NU/NU Mice, the Beijing Charles River Experimental Animal Technology Co., Ltd., Beijing, China). Briefly, 2×10^6 cells were subcutaneously grown in mice (n = 6 per group). After tumor formation, the tumor size was observed and recorded every 5 days. Then, tumors were removed, photographed for documentation, and embedded in paraffin for IHC assays.

For the tail vein injection method for lung metastasis model, six-week-old male NU/NU nude mice were randomly divided into five groups (n = 6 per group). 2×10^6 MHCC-97H cells were resuspended in 100 μL PBS and injected into the tail vein of the mice. Five weeks after injection, the mice were sacrificed. The lung tissues were collected and fixed in paraformaldehyde.

For the functional analysis associated with CPPtat-V1 peptide (YGRKKRRQRRR IAGDSSRTSLVSTIAG) and CPPtat-V2 peptide (YGRKKRRQRRR IAGDSSRTSLVSTIAG) (GenScript Biotech Corporation, Nanjing, China) 1×10^6 MHCC-97H cells were resuspended with 50 μL Matrigel (Cat #354234, Corning, USA) and 50 μL PBS and injected into BALB/c-nude mice (4 to 6 weeks old, male, 20–25 g; obtained from Chongqing Ensville Biotechnology Co., Ltd., Chongqing, China). Ten days after tumor colonization, 10 mg/kg of CPPtat-V1 peptide or CPPtat-V2 peptide was injected intraperitoneally every three days. After 25 days post implantation, mice were sacrificed, and liver tissues were harvested for histological examination.

Immunohistochemistry (IHC)

After treated with normal goat serum (ZSGB-BIO, Beijing, China) for 1 h, tissue sections

were incubated with primary GFAT1 antibody (dilution 1: 500) (Proteintech, CA, USA) or primary VEZF1 antibody (dilution 1: 100) (Santa Cruz Biotechnology, sc-365560, USA) at 4 °C overnight. After washing 3 times with PBS for 5 min each, sections were treated with a secondary goat-anti-rabbit or goat-anti-mouse IgG antibody (ZSGB-BIO) for 30 min. The nuclei were stained with hematoxylin for 3 min. The stained sections were then scanned with the MoticEasyScanner (Motic, Hong Kong, China) and images were acquired using Caseviewer (3DHISTECH, Budapest, Hungary). Information on the microarray of immune tissues from HCC patients is presented in Supplementary Table S5.

Statistical analysis

Statistical analysis was performed with GraphPad Prism 8.0 software (GraphPad Software, San Diego, CA, USA). Data were presented as the mean ± standard deviation (SD). Tests used to examine the differences between groups include Student's t-test, one-way ANOVA and $\chi 2$ test. Pearson Correlation Coefficient (r) was used to test the linear correlation. P-values < 0.05 were considered statistically significant. *P < 0.05, **P < 0.01, ***P < 0.001.

References

1 Xiang J, Chen C, Liu R, Gou D, Chang L, Deng H *et al.* Gluconeogenic enzyme PCK1 deficiency promotes CHK2 O-GlcNAcylation and hepatocellular carcinoma growth upon glucose deprivation. *J Clin Invest* 2021; **131**: e144703, 144703.

2 DeCaprio J, Kohl TO. Chromatin Immunoprecipitation. *Cold Spring Harb Protoc* 2020; **2020**: pdb.prot098665.

Supplementary Figures and Figure Legends

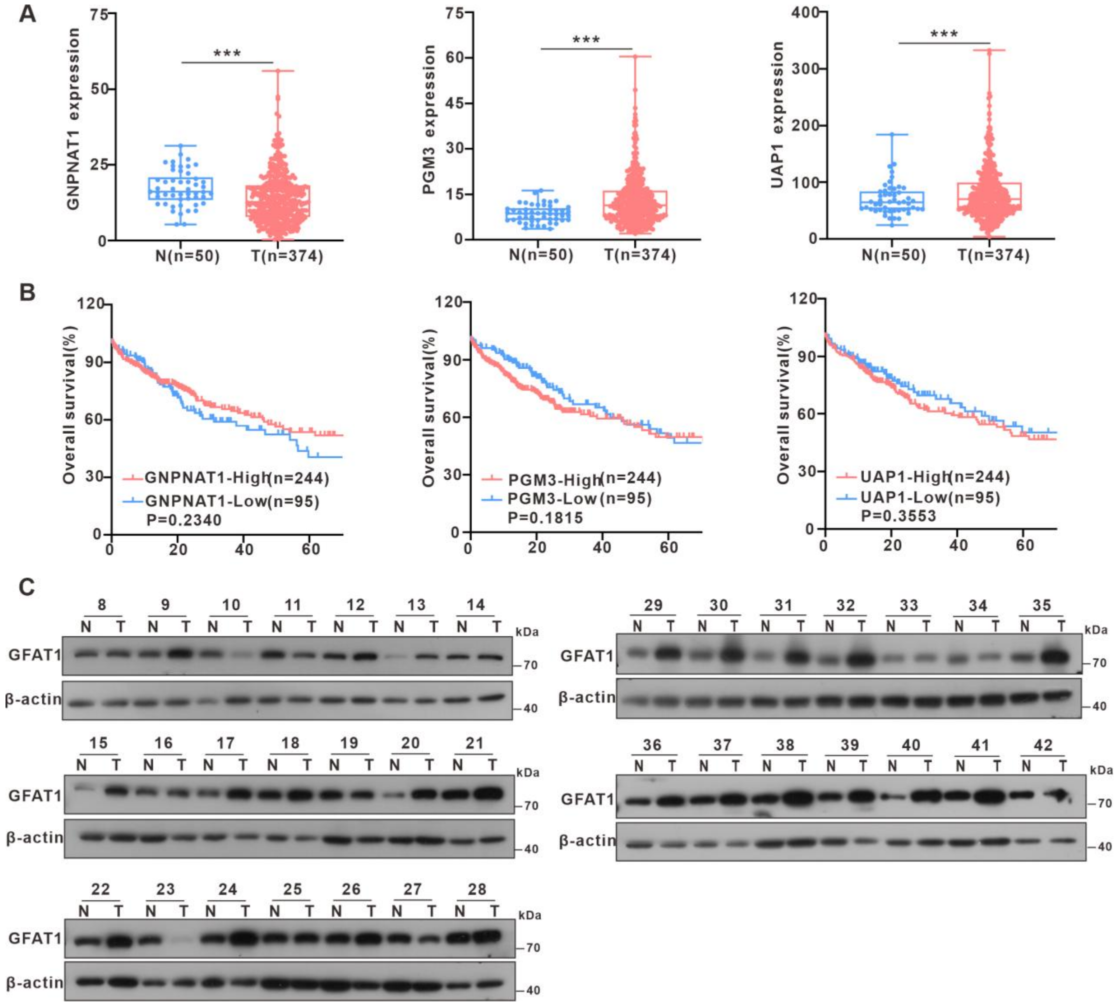

Fig. S1 Expression and prognosis of HBP metabolic enzymes in HCC. (A) Analysis of mRNA expression level of *GNPNAT1, PGM3* and *UAP1* in HCC and noncancerous tissues based on TCGA database. (B) Kaplan–Meier survival analysis of *GNPNAT1, PGM3* and *UAP1* depicting the overall survival (OS) of patients with HCC from the TCGA . (C) Western blot was used to detect the protein expression of GFAT1 in 42 pairs of HCC tissues and the adjacent nontumor tissues of HCC patients (N non-tumor tissue, T tumor tissue).

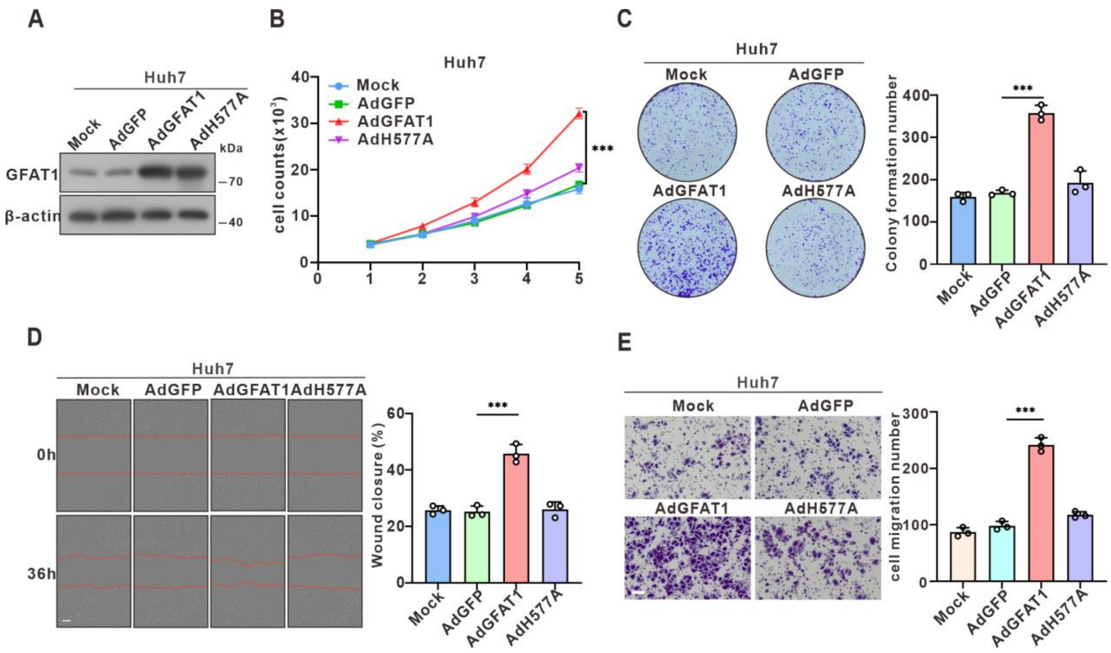

Fig. S2 GFAT1 promotes hepatoma cell proliferation and tumorigenesis. (A) Western blot was used to detect the protein expression of GFAT1 in Huh7 infected with AdGFAT1-WT or GFAT1-H577A mutant adenovirus. (B) Growth curves of hepatoma cells. (C) Colony formation capacity of Huh7 cells. (D, E) Representative and quantified results of the wound-healing assays (D) and transwell (E) in Huh7 cells. Statistical analysis was shown as mean ± SD (n = 3). One-way ANOVA followed by the Tukey test, *P < 0.05, **P < 0.01, ***P < 0.001. Scale bar: 50 µm.

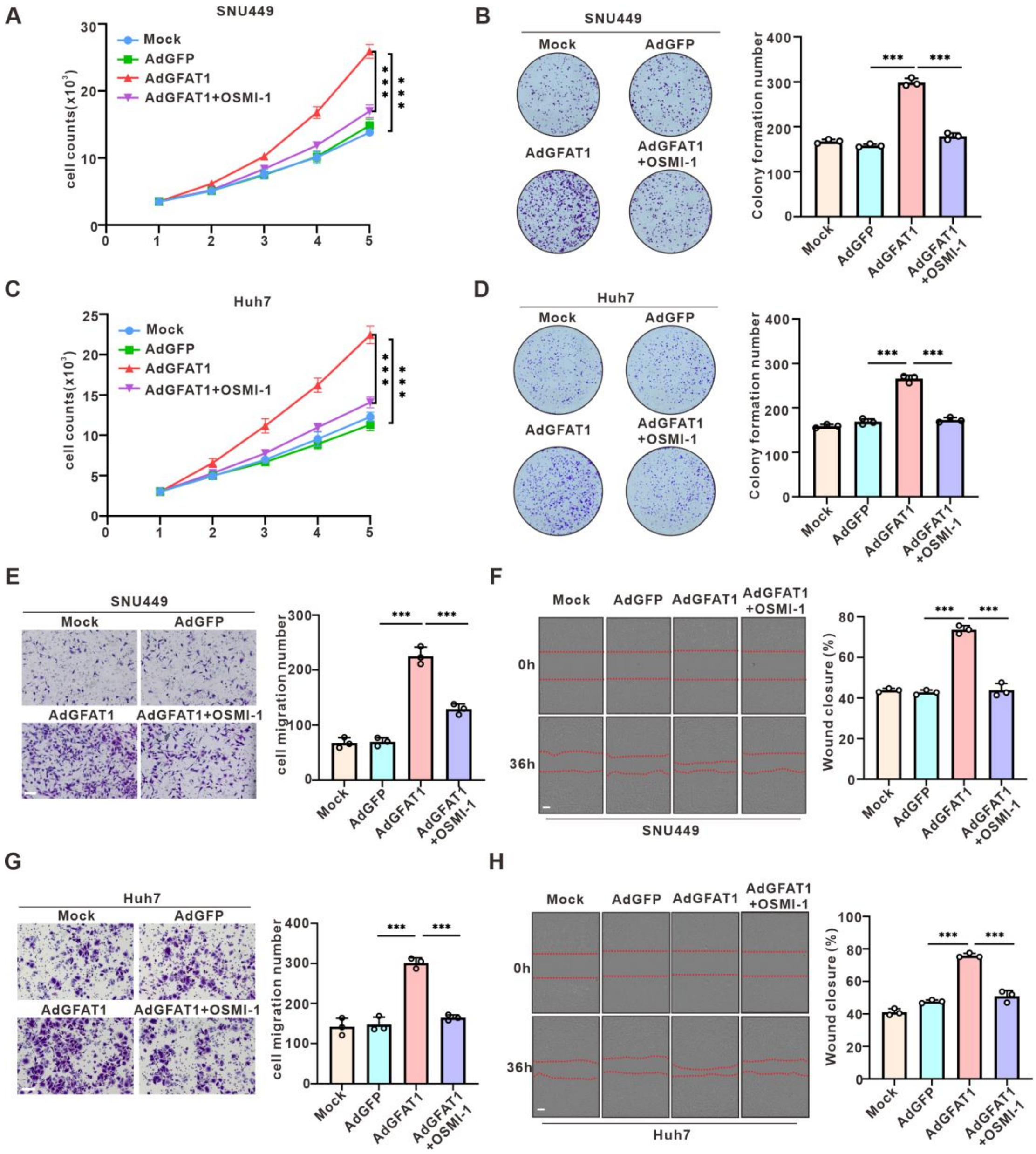

Fig. S3 GFAT1 promotes hepatoma cell proliferation and invasion by enhancing O-GlcNAcylation. (A, C) Growth curves of hepatoma cells. (B, D) Colony formation capacity of SNU449 and Huh7 cells. (E, G) Representative and quantified results of the transwell in SNU449 and Huh7 cells. Scale bar: 20 μm. (F, H) wound-healing assays in SNU449 and Huh7 cells. Scale bar: 50 μm. Statistical analysis was shown as mean ± SD (n = 3). One-way ANOVA followed by the Tukey test, *P < 0.05, **P < 0.01, ***P < 0.001.

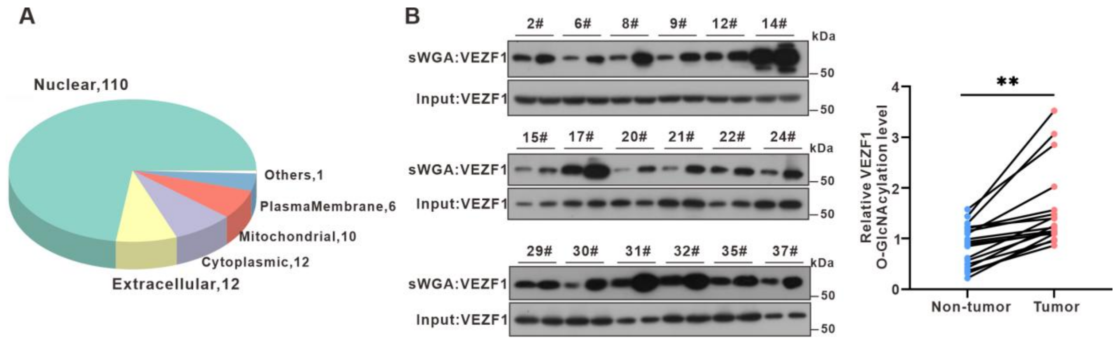

Fig. S4 O-GlcNAcylation occurs on VEZF1. (A) Pie chart of subcellular localization of proteins belonging to O-GlcNAcylated peptides. (B) sWGA pull-down assays were carried out in HCC patients with high GFAT1 expression (the expression refers to Fig. S1, n=18), and VEZF1 O-GlcNAcylation levels were quantified by ImageJ and analyzed with two-tailed paired t-test. **P < 0.01.

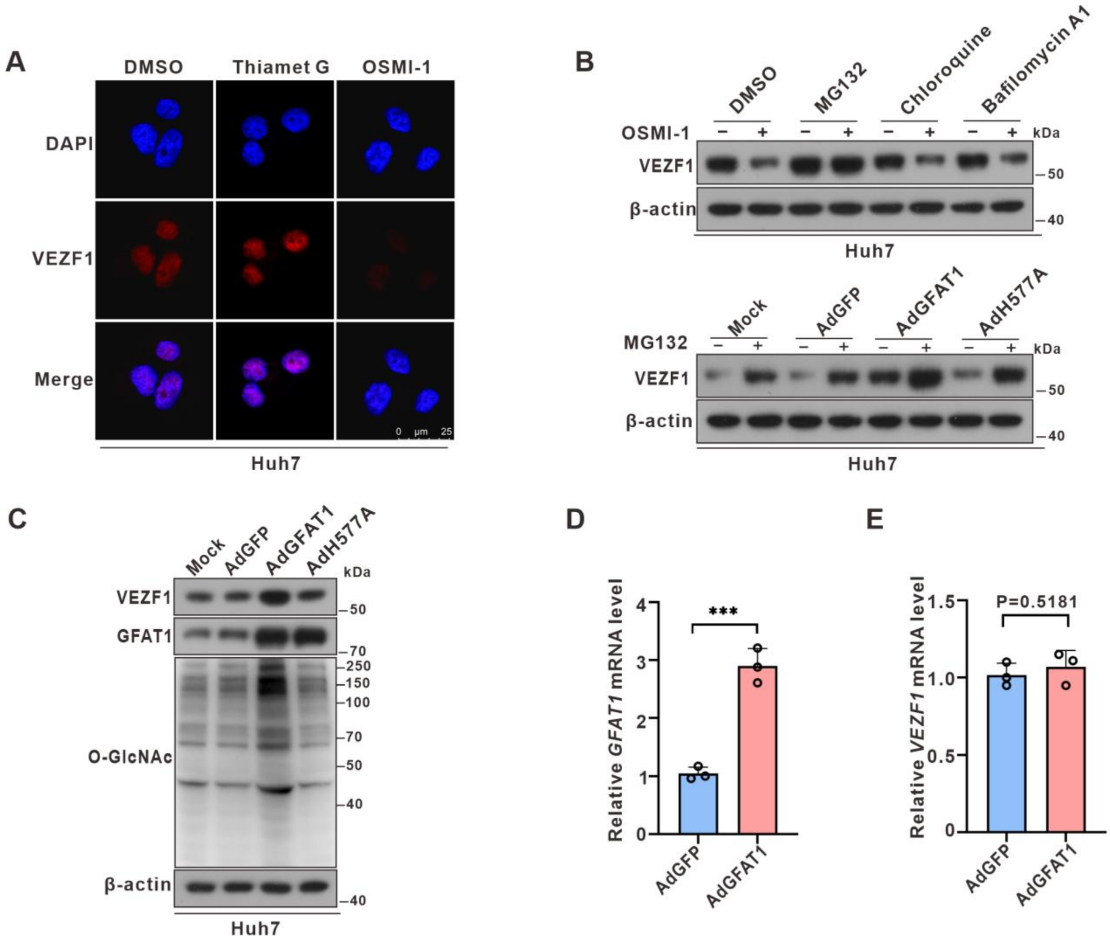

Fig. S5 O-GlcNAcylation did not affect the Nucleocytoplasmic localization of VEZF1 but protein stability. (A) VEZF1 subcellular localization in Huh7 cellswas detected by immunofluorescence staining. Cells were treated with DMSO, 50μM Thiamet G, or 25μM OSMI-1 12 h before experimentation. (B) Huh7 cells were treated with 10 μM MG132, 50 μM chloroquine (CQ) or 100 μM bafilomycin A1 (BafA1) for 6 h before treatment with 25 μM OSMI-1. (C) The protein expression levels of VEZF1 were measured by immunoblotting in parental or GFAT1-OE cells (D, E) The mRNA expression levels of GFAT1 and VEZF1 were measured by qRT-PCR. Statistical analysis was shown as mean ± SD (n = 3). ***P < 0.001.

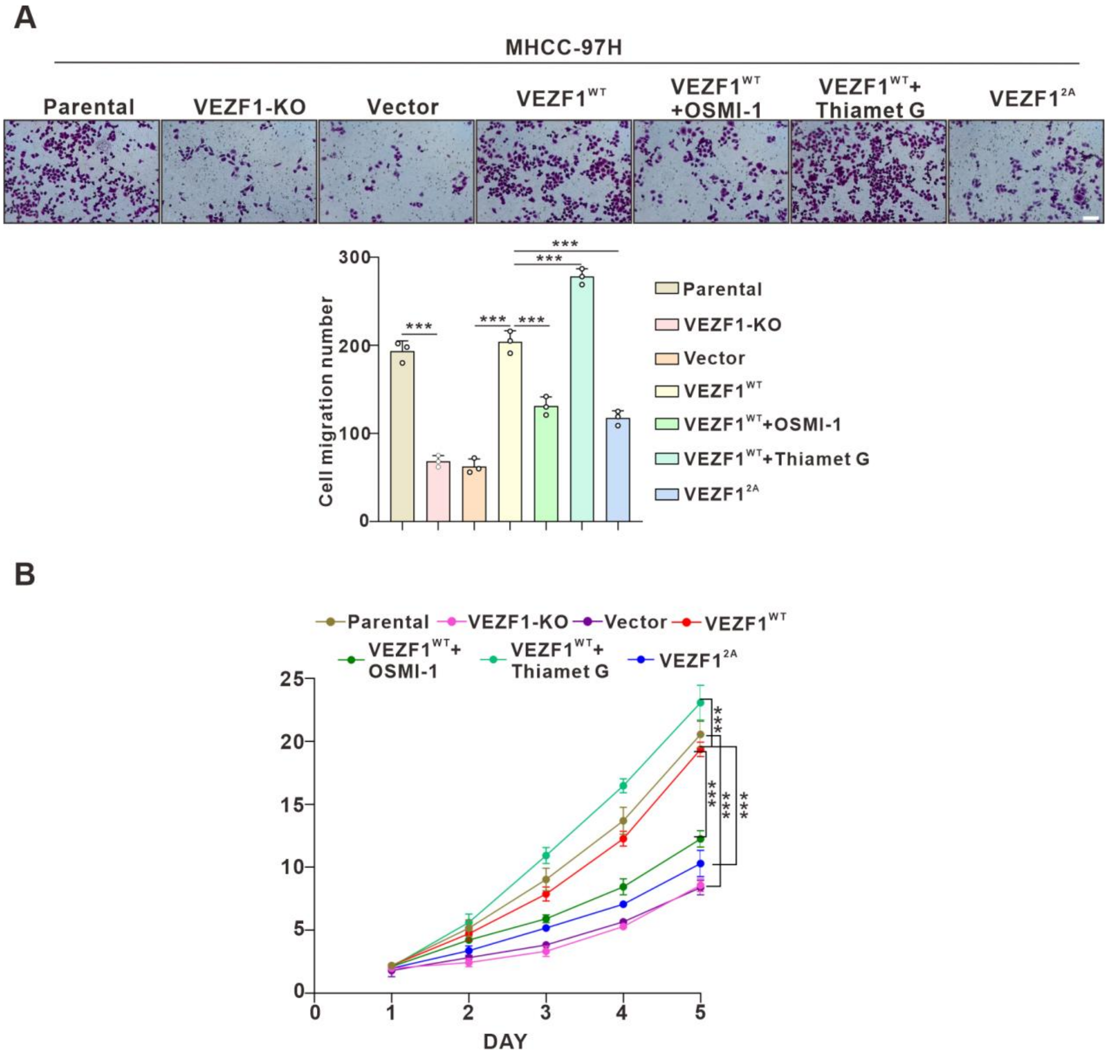

Fig. S6 VEZF1 O-GlcNAcylation promotes HCC proliferation. (A) Representative and quantified results of transwell assays in MHCC-97H cells. (B) Growth curves of MHCC-97H cells (n = 3 independent experiments). Statistical analysis was performed using one-way ANOVA with Tukey’s test, ***$P < 0.001$.

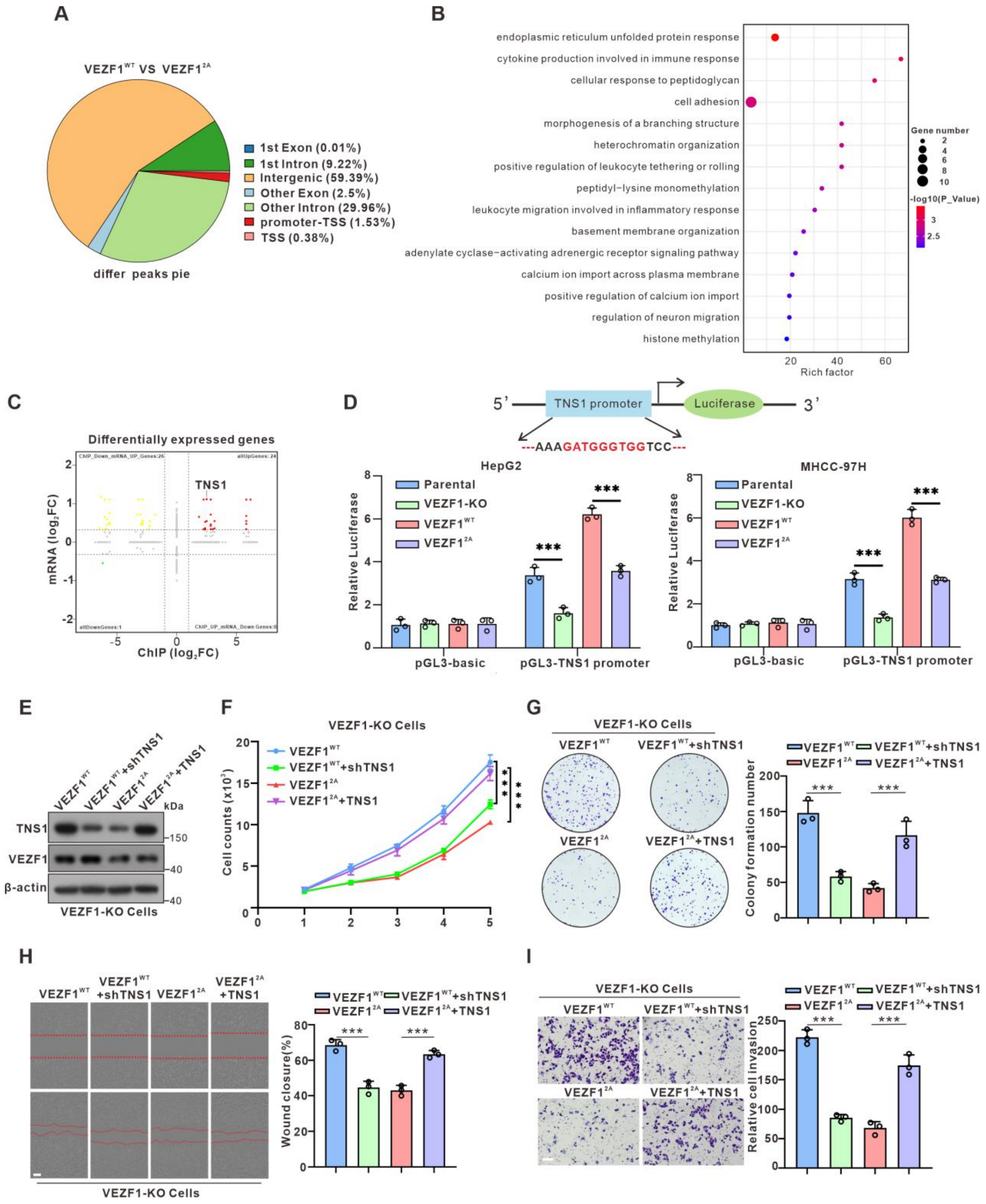

Fig. S7 VEZF1 O-GlcNAcylation promotes HCC proliferation by promoting the transcription of TNS1. (A) The differential peak distribution of VEZF1^{WT} and VEZF1^{2A} was analyzed by Chip-seq. (B) GO analysis of differentially expressed genes in the promoter region between VEZF1^{WT} and VEZF1^{2A}. (C) Joint analysis of CHIP-seq and RNA-seq. (D) The dual-luciferase reporter assay for the promoter region binding affinity of TNS1 in HepG2 and MHCC-97H cells, n = 3 biologically

independent experiments. Statistical analysis was shown as mean ± SD (n = 3). (E) The protein expression levels of VEZF1 and TNS1 in VEZF1-KO cells. (F) Growth curves of MHCC-97H VEZF1-KO cells (n = 3 independent experiments). (G) Colony formation capacity and quantified results of VEZF1-KO cells (n = 3 independent experiments). (H) Representative and quantified results of the wound-healing assays in MHCC-97H VEZF1-KO cells. Scale bar: 50 μm. (I) Representative and quantified results of transwell assays in MHCC-97H VEZF1-KO cells. Scale bar: 20 μm. Two-way ANOVA (D), One-way ANOVA followed by the Tukey test (E-H), $*P < 0.05$, $**P < 0.01$, $***P < 0.001$.

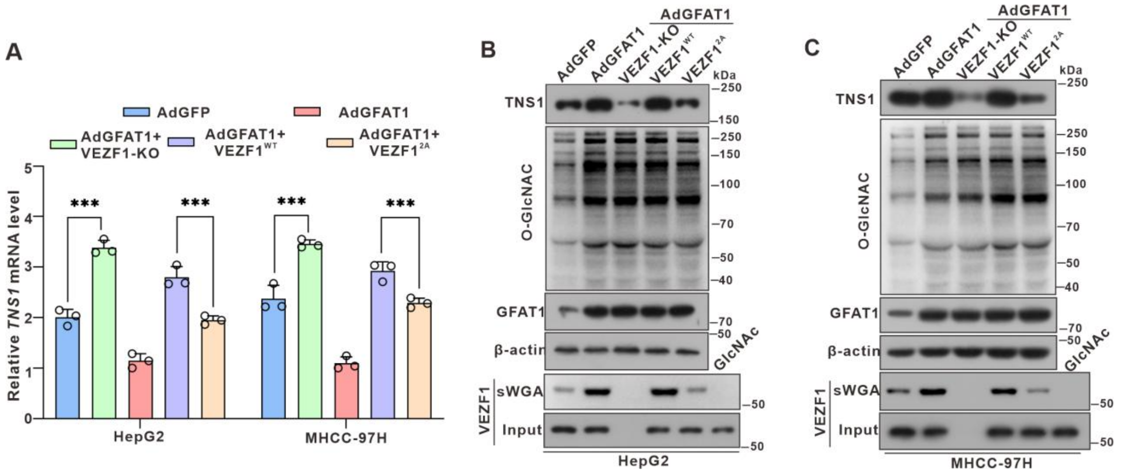

Fig. S8 GFAT1 promotes VEZF1 O-GlcNAcylation and thus promotes TNS1 transcription. (A) qPCR analysis of TNS1 expression in HepG2 and MHCC-97H Statistical analysis was shown as mean ± SD (n = 3). One-way ANOVA followed by the Tukey test, ***P < 0.001. (B, C) Western blotting was used to detect the protein expression levels of GFAT1 and TNS1; the O-GlcNAcylation level of VEZF1.

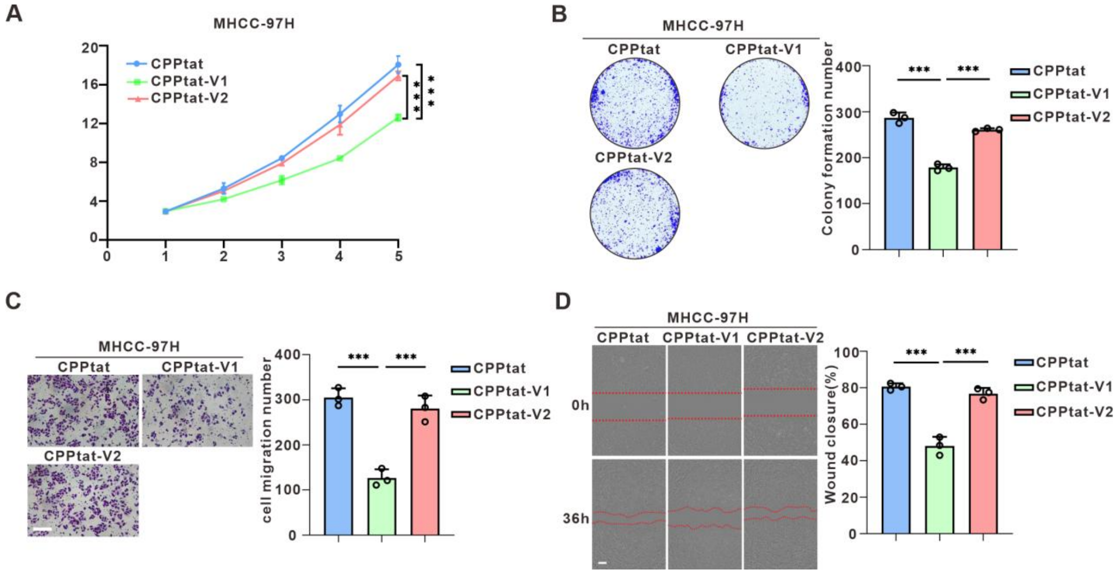

Fig. S9 CPPtat-V1 inhibited the progression of liver cancer. MHCC-97H cells were treated with 10μM CPPtat, CPPtat-V1 and CPPtat-V2 to observe the proliferation and migration ability of HCC. (A) Growth curves of hepatoma cells. (B) Colony formation capacity of MHCC-97H cells. (C) Representative and quantified results of the transwell assays in MHCC-97H cells. Scale bar: 20 μm. (D) Representative and quantified results of wound-healing assays in MHCC-97H VEZF1-KO cells. Scale bar: 50 μm. Statistical analysis was shown as mean ± SD (n = 3). One-way ANOVA followed by the Tukey test, *P < 0.05, **P < 0.01, ***P < 0.001.

Supplementary Table

Table. S1 Primer sequences of gene cloning and mutagenesis were used in this study.

Plasmids	Forward (5'-3')	Reverse (5'-3')
pAdTrack-TO4-GFAT1	TGGGGTACCATGGGCTGTGGTATATTTGCTTACTTAAA	CCCAAGCTTTCACTCTACAGTCACAGATTTGG
pAdTrack-TO4-H577A	GTGAATTGAAAGCTGGCCCTCTGGCTTTGG	CCAGAGGGCCAGCTTTCAATTCACCAGCAA
pSEB-3Flag-OGT	ATAGCGGCCGCACCATGGCGTCTTCCGTGGGC	CGGGGTACCTGCTGACTCAGTGACTTCAACAGG
pBu-3HA-VEZF1	TATGGTACCATGGAGGCCAACTGGACC	GGAAGATCTCTCCAAGGCGGTGATGTAGG
VEZF1-1-167aa	TATGGTACCATGGAGGCCAACTGGACC	GGAAGATCTCTACTGGGTTTCTTGACAGACTG
VEZF1-168-361aa	TATGGTACCATGGGCAAGCCTGTCAAGAAGAACCA	GGAAGATCTCTCCCTGGCCAGCTTGTCA
VEZF1-362-521aa	TATGGTACCATGGGCAAGCAAGTAGAAACACTGAGA	GGAAGATCTCTCCAAGGCGGTGATGTAGG
pBu-3HA-T108A&T110A	AAGAAAGCCCCCGCCACGGTGGTTCCCCTT	GGGAACCACCGTGGCGGGGGCTTTCTTTGG
pBu-3HA-S117A&S118A	CCTTATCGCTGCCATCGCTGGGGACAGCA	CCCAGCGATGGCAGCGATAAGGGGAACCA
pBu-3HA-S123A&S124A	CGCTGGGGACGCCGCCCGAACTTCGTTGGTCTCG	ACCAACGAAGTTCGGGCGGCGTCCCCAGCGATGG
pBu-3HA-S123A	CGCTGGGGACGCCAGCCGAACTTCGTTGGTC	AACGAAGTTCGGCTGGCGTCCCCAGCGATGG
pBu-3HA-S124A	CTGGGGACAGCGCCCGAACTTCGTTGGTCTC	CAACGAAGTTCGGGCGCTGTCCCCAGCGATG
pAdTrack-TO4-VEZF1-3HA	TATGGTACCATGGAGGCCAACTGGACC	CCGCTCGAGGGTACGCGTTCATTAGGCGTA
pAdTrack-TO4-S123A&S124A-3HA	CGCTGGGGACGCCGCCCGAACTTCGTTGGTCTCG	CCGCTCGAGGGTACGCGTTCATTAGGCGTA
pGL3-Basic-TNS1	AGGGGTACCCCAACAAGATTACTGATAGATTACA	GGAAGATCTGGAGGTGGGGAAGGTATC

Table. S2 Sequences of shRNAs and sgRNAs in this study.

Target gene	Forward (5'-3')	Reverse (5'-3')
shGFAT1#1	TGGAGAGAGTTATCCAACAAT TCAAGAGATTGTTGGATAACT CTCTCCTTTTTTC	TCGAGAAAAAAGGAGAGAGTT ATCCAACAATCTCTTGAATTGTT GGATAACTCTCTCCA
shGFAT1#2	TGGAAGTACTGAGCATGGATT TCAAGAGAATCCATGCTCAGT ACTTCCTTTTTTC	TCGAGAAAAAAGGAAGTACTG AGCATGGATTCTCTTGAAATCCA TGCTCAGTACTTCCA
shGFAT1#3	TGGTTTGAAGGATCACATAAT TCAAGAGATTATGTGATCCTT CAAACCTTTTTTC	TCGAGAAAAAAGGTTTGAAGG ATCACATAATCTCTTGAATTATG TGATCCTTCAAACCA
sgGFAT1#1	CACCGGAGAGAGTTATCCAA CAAT	AAACATTGTTGGATAACTCTCTC C
sgGFAT1#2	CACCGATGTTGCAGCAGTAGT GGA	AAACTCCACTACTGCTGCAACA TC
sgGFAT1#2	CACCGGAACTCCAGCAGATC ATGA	AAACTCATGATCTGCTGGAGTT CC
sgVEZF1#1	CACCGATTGAGATGGTACACA TCT	AAACAGATGTGTACCATCTCAA TC
sgVEZF1#2	CACCGTCGGCCGCCGCAGCC ATGG	AAACCCATGGCTGCGGCGGCCG AC
sgVEZF1#3	CACCGAACAGGAACGCGGTC CAGT	AAACACTGGACCGCGTTCCTGT TC
shTNS1#1	TGCAACTACCTGCTGTTCAAC CTTCAAGAGAGGTTGAACAG CAGGTAGTTGCTTTTTTC	TCGAGAAAAAAGCAACTACCT GCTGTTCAACCTCTCTTGAAGG TTGAACAGCAGGTAGTTGCA
shTNS1#2	TGCAGGATAGGAGTTGTCATC GTTCAAGAGACGATGACAAC TCCTATCCTGCTTTTTTC	TCGAGAAAAAAGCAGGATAGG AGTTGTCATCGTCTCTTGAACG ATGACAACTCCTATCCTGCA
shTNS1#3	TGCAGGTCTTACTCACCTTAT GTTCAAGAGACATAAGGTGA GTAAGACCTGCTTTTTTC	TCGAGAAAAAAGCAGGTCTTAC TCACCTTATGTCTCTTGAACATA AGGTGAGTAAGACCTGCA

Table. S3 Sequence for quantitative real-time reverse transcription PCR

Target gene	Forward (5'-3')	Reverse (5'-3')
GFAT1	CCAGTCCTGTCAATAGCCACC	CACAAGTGCAAAAGCACCTTC
VEZF1	CACAGGGATCAAGTTGGTGTC	TTGGTGCCCGAGGAAGAT
TNS1	AACCGAGGCAGGATAGGAG	TGGTAGATGCGGAGAAATGG
TNS1 ChIP-qPCR #1	CCAACAGCACAGGGAACAGA	TGCCCAGATACCAGAGTCAGAACA
TNS1 ChIP-qPCR #2	TTGTACGCTGCCCACCC	CCATCTGTTCCCTGTGCTGTT
TNS1 ChIP-qPCR #3	ATCGTGGGACCAGGAGAATT	TGGTAAGGAGCCAGGGTGT
TNS1 ChIP-qPCR #4	CAGGGTGGAAGCAGAGGTG	GGCAGGTGCCCAGGAAA
TNS1 ChIP-qPCR #5	CCATCGTGGGACCAGGAGA	TTGGGCTCAGGCTGGGTGT

Table. S4 Information of antibodies

Antibodies	Source	Catalogue #
Rabbit polyclonal, GFAT1	Proteintech	14132-1-AP
Mouse monoclonal, O-Linked N-AcetylGlucosamine	Abcam	Ab2739
Rabbit monoclonal, OGT	Abcam	Ab177941
Mouse monoclonal, HA tag	Invitrogen	26183
Mouse monoclonal, FLAG M2	Sigma-aldrich	F1804
VEZF1 antibody	Santa Cruz Biotechnology	sc-365560
Goat anti-Mouse secondary antibodies/FITC	ZSGB-BIO	ZF-0312
Goat anti-Rabbit secondary antibodies/FITC	ZSGB-BIO	ZF-0322
Goat anti-Mouse secondary antibodies/TRITC	ZSGB-BIO	ZF-0316
Goat anti-Rabbit secondary antibodies/TRITC	ZSGB-BIO	ZF-0313
Mouse monoclonal，β-actin	ZSGB-BIO	TA-09
His tag	Proteintech	66005-1-Ig
PCNA	Proteintech	10205-2-AP
TNS1	proteintech	20054-1-AP

Table. S5 Clinical characteristics of HCC patients

	gender	IHC Score T	IHC Score N	age	TNM stage	Survival or not (1 death)	Survival time (months)
B15,B16	male	12	8	46	I	0	74.73333333
C05,C6	male	12	6	49	IV	1	65.76666667
C09,C10	male	12	8	56	IV	1	45.3
C13,C14	male	12	8	44	I	0	69.96666667
D07,D8	female	12	6	50	III	1	36
D09,D10	male	12	6	55	III	0	103.8
D17,D18	female	12	6	33	IV	1	15.13333333
E11,E12	male	12	6	63	IV	1	36.1
E17,E18	male	12	8	47	II	0	84.76666667
F07,F8	male	12	8	55	IV	1	33.86666667
F13,F14	male	12	6	61	IV	1	16.86666667
F17,F18	male	12	8	48	II	1	56.03333333
I05,I6	male	12	10	47	II	0	87.63333333
D13,D14	male	11	9	21	III	1	37.3
F15,F16	male	10	8	41	IV	1	32.23333333
G07,G8	male	10	8	48	II	0	70
G17,G18	male	10	7	41	II	0	75.46666667
H05,H6	male	10	5	43	III	1	29.5
I13,I14	male	10	6	51	III	1	26.9
J09,J10	female	10	8	45	III	1	15.8
B03,B4	male	9	6	49	IV	1	66.93333333
B09,B10	male	9	8	51	I	1	31.43333333
C07,C8	female	9	4	45	I	0	65.93333333
C11,C12	male	9	6	55	IV	1	11.8
C15,C16	male	9	6	33	I	1	31.93333333
D03,D4	male	9	4	49	III	1	9.866666667
D05,D6	male	9	4	48	I	1	23.33333333

D11,D12	male	9	6	45	IV	1	20.6
E01,E2	male	9	8	56	III	1	4.766666667
E03,E4	male	9	6	53	IV	1	58.73333333
E15,E16	male	9	6	42	IV	1	21.56666667
F9,F10	male	9	6	48	IV	1	11.83333333
G05,G6	male	9	8	28	III	1	38.96666667
I15,I16	male	9	9	46	III	1	4.2
I17,I18	male	9	5	50	IV	1	14.06666667
J01,J2	male	9	6	59	IV	1	48.1
B01,B2	male	8	8	40	II	1	32.63333333
B13,B14	female	8	8	50	I	0	79.1
D01,D2	male	8	9	59	III	1	14.66666667
E05,E6	male	8	9	48	II	1	45.13333333
E07,E8	male	8	9	42	IV	1	21.33333333
G13,G14	male	8	7	59	II	0	68.7
I03,I4	male	8	7	73	IV	1	14.8
E09,E10	male	7	8	41	II	0	107.8333333
F11,F12	male	7	4	64	III	0	78.9
G11,G12	male	7	8	61	II	0	70.3
H03,H4	male	7	6	54	II	0	70.73333333
A05,A6	male	6	4	58	I	0	71.5
A09,A10	male	6	4	47	I	0	77.23333333
A13,A14	male	6	6	60	I	0	69.73333333
B07,B8	male	6	6	57	I	0	74.9
B11,B12	male	6	8	45	I	0	74.83333333
B17,B18	male	6	6	42	III	0	66.06666667
C03,C4	male	6	8	43	I	0	66
F03,F4	male	6	4	40	IV	1	98.83333333
G03,G4	female	6	4	50	IV	0	69.83333333
G9,G10	male	6	9	65	II	0	70.93333333

H01,H2	male	6	9	73	II	0	75.3
H15,H16	male	6	6	33	II	0	78.16666667
I01,I2	male	6	6	25	III	1	28.8
I11,I12	male	6	6	50	IV	1	26.4
J03,J4	male	6	4	41	III	0	61.1
B05,B6	male	5	6	42	I	0	74
G15,G16	male	5	8	51	III	0	80.16666667
H07,H8	male	5	6	55	III	1	73.4
I09,I10	male	5	7	63	IV	1	54.5
J11,J12	male	5	4	26	III	1	7.9
A03,A4	female	4	6	52	I	0	82.53333333
A11,A12	female	4	4	44	I	0	65.03333333
C01,C2	female	4	8	60	I	0	73.06666667
C17,C18	male	4	6	43	I	0	69.23333333
D15,D16	male	4	9	30	II	1	25.96666667
E13,E14	male	4	8	39	II	0	81.56666667
F05,F6	male	4	6	54	III	1	46.33333333
G01,G2	male	4	6	30	II	1	63.56666667
H09,H10	male	4	8	16	III	1	31.13333333
H11,H12	male	4	6	64	III	0	80.4
H13,H14	male	4	6	49	III	1	22
H17,H18	male	4	2	70	II	0	75.86666667
J05,J6	male	4	4	41	II	1	41.23333333
J07,J8	male	4	3	59	IV	1	15.26666667
I07,I8	male	3	5	62	IV	1	43.43333333
A07,A8	male	2	4	45	I	0	89.16666667
F01,F2	male	2	4	34	II	0	85.66666667
J13,J14	male	2	0	54	IV	1	66.46666667
