# Supplementary material for: GFAT1 promotes the progression of hepatocellular carcinoma via enhancing the O-GlcNAcylation of VEZF1
Source: Cell Death Dis. 2025 Aug 26;16(1):647. doi: 10.1038/s41419-025-07975-5 (PMC12381164; doi:10.1038/s41419-025-07975-5)
Supplement: Supplementary file 2 — WB Data [file 41419_2025_7975_MOESM2_ESM.pdf]

Fig1E

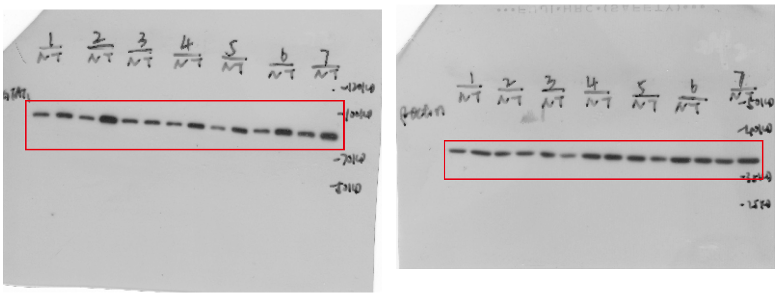

Fig1G

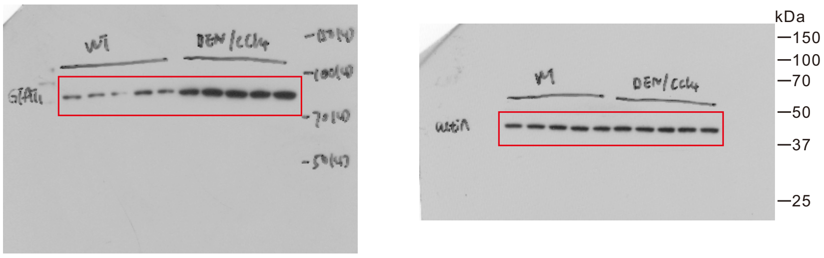

Fig1H

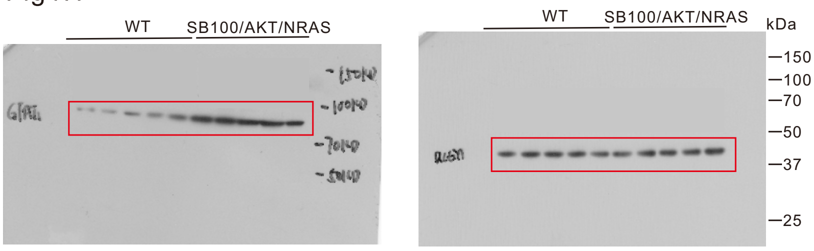

Fig2A

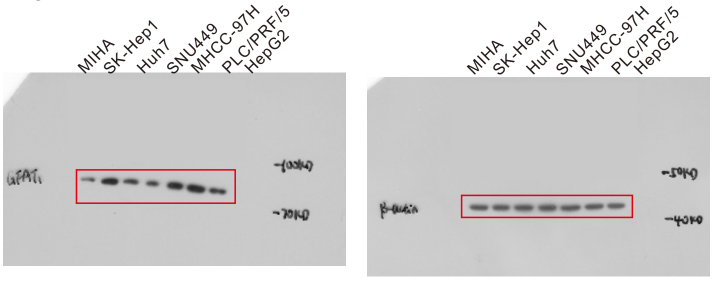

Fig2B

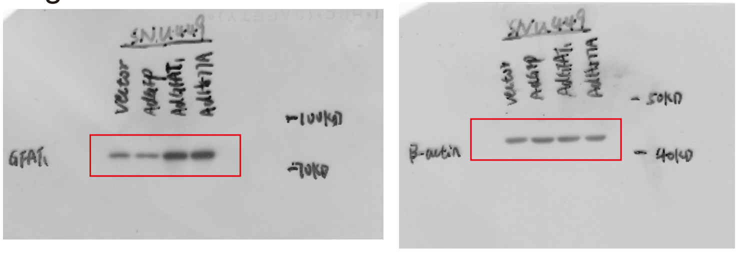

Fig2C

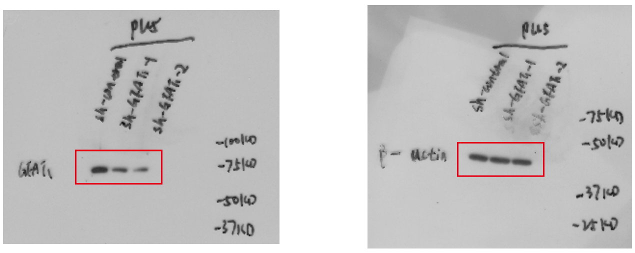

Fig3G

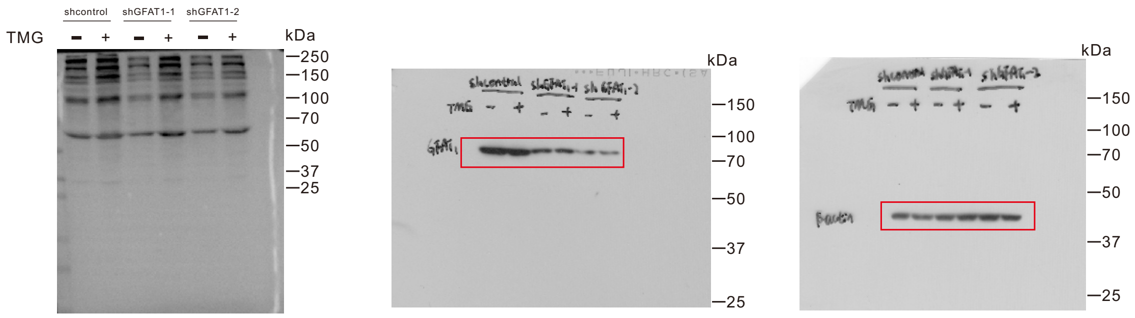

Fig3H

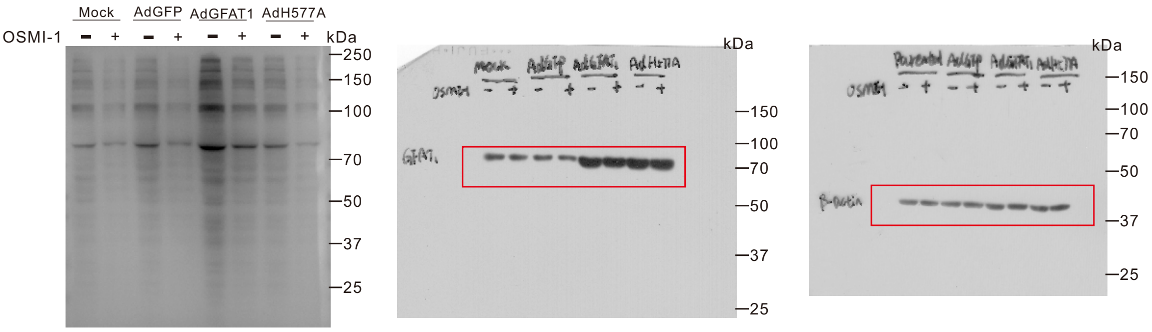

Fig4C

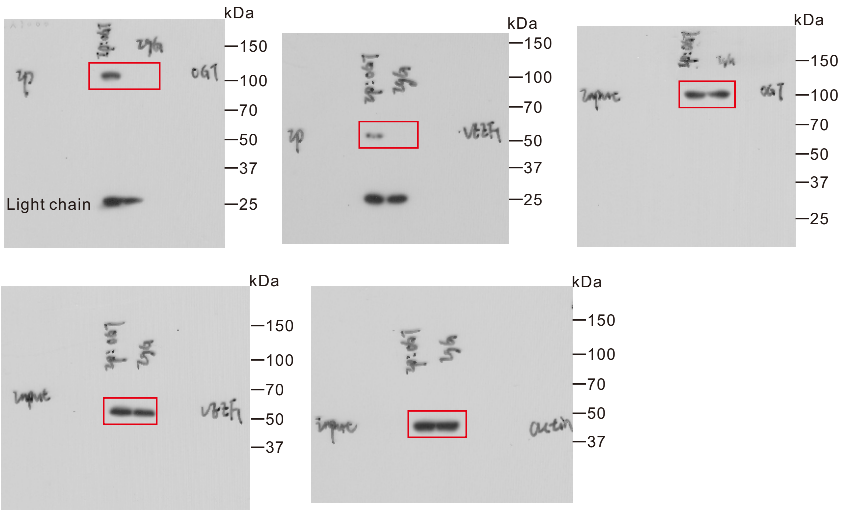

Fig4D

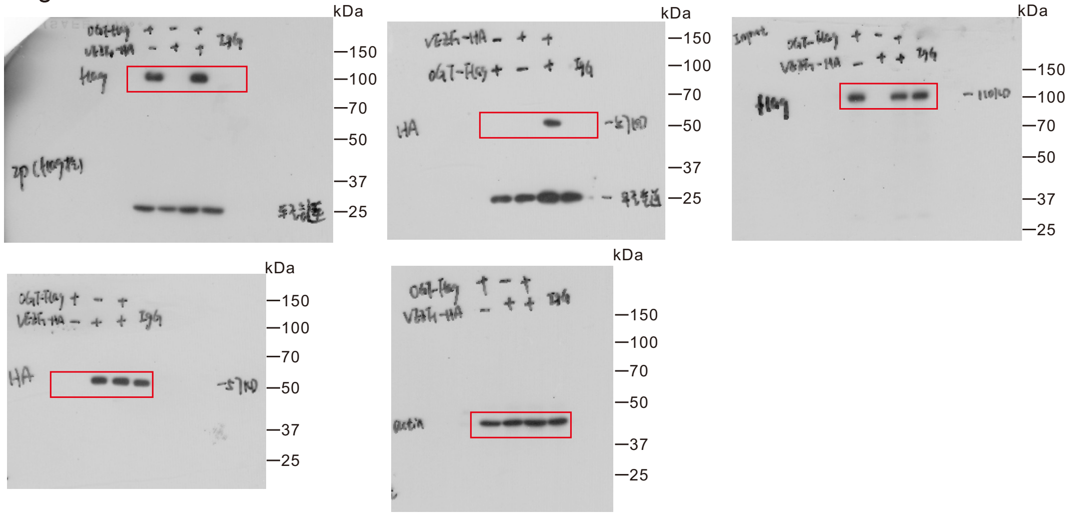

Fig4E

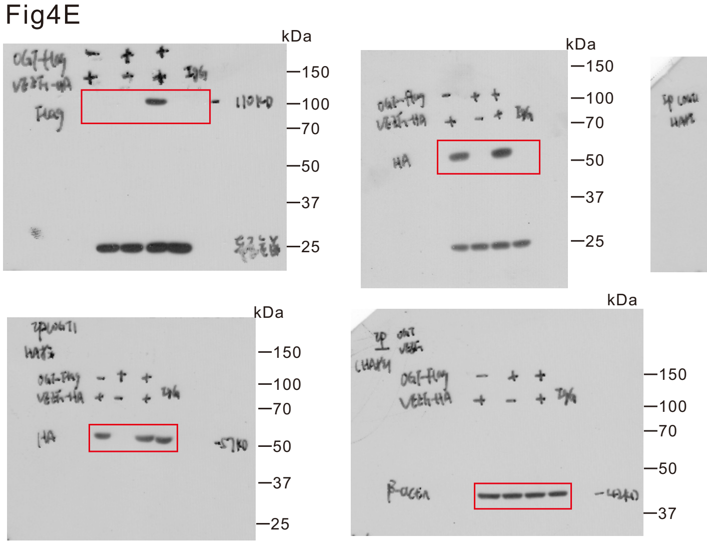

Fig4H

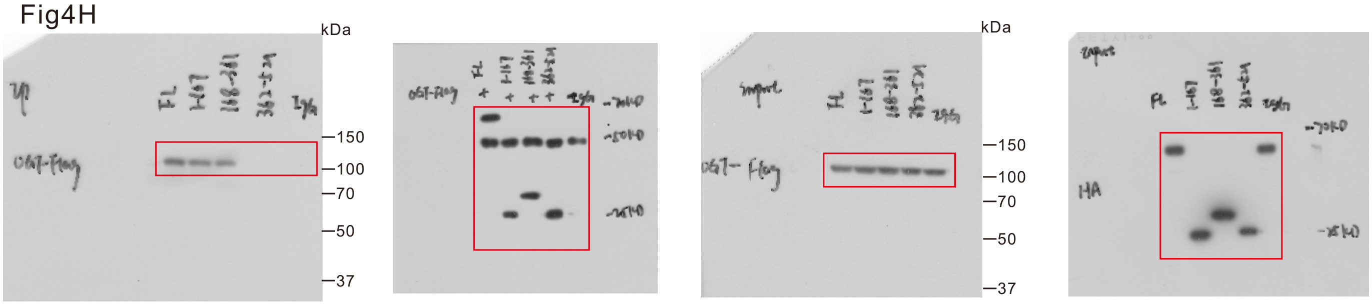

Fig4I

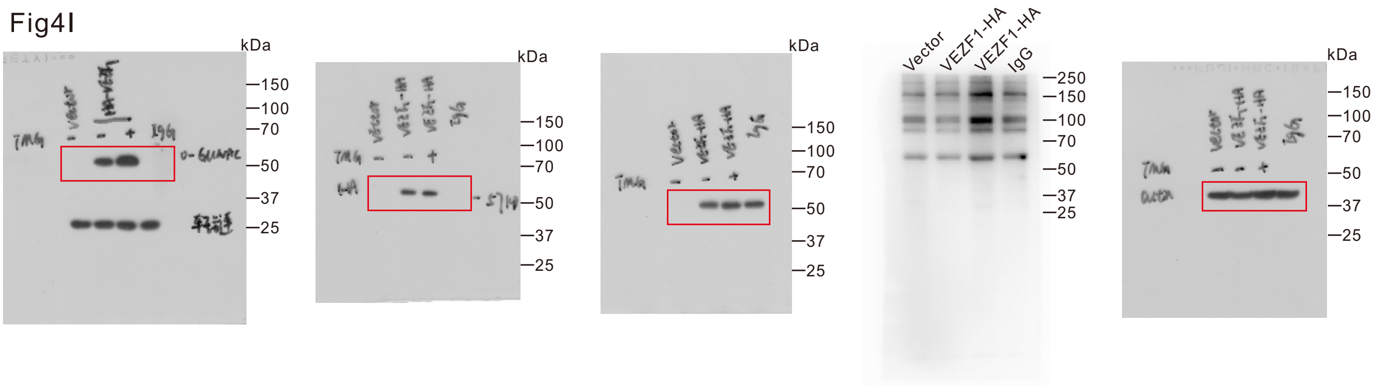

Fig4J

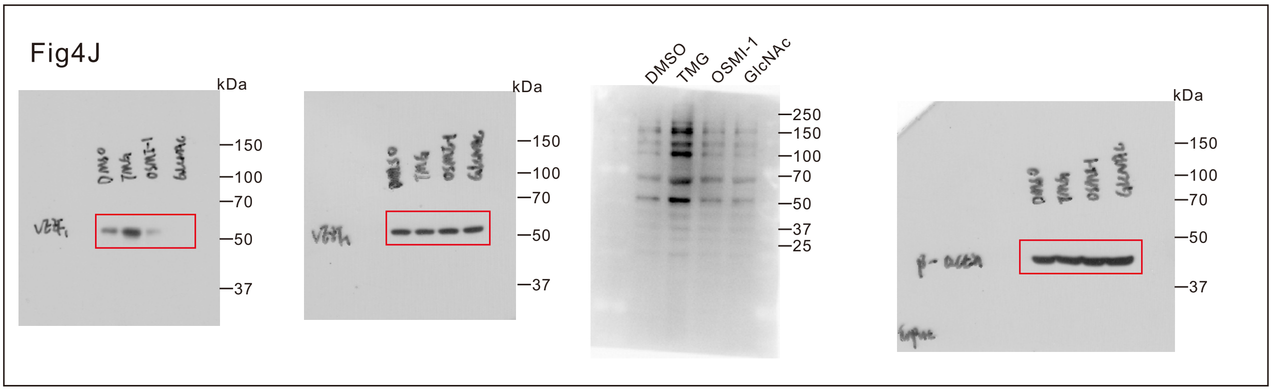

Fig4K

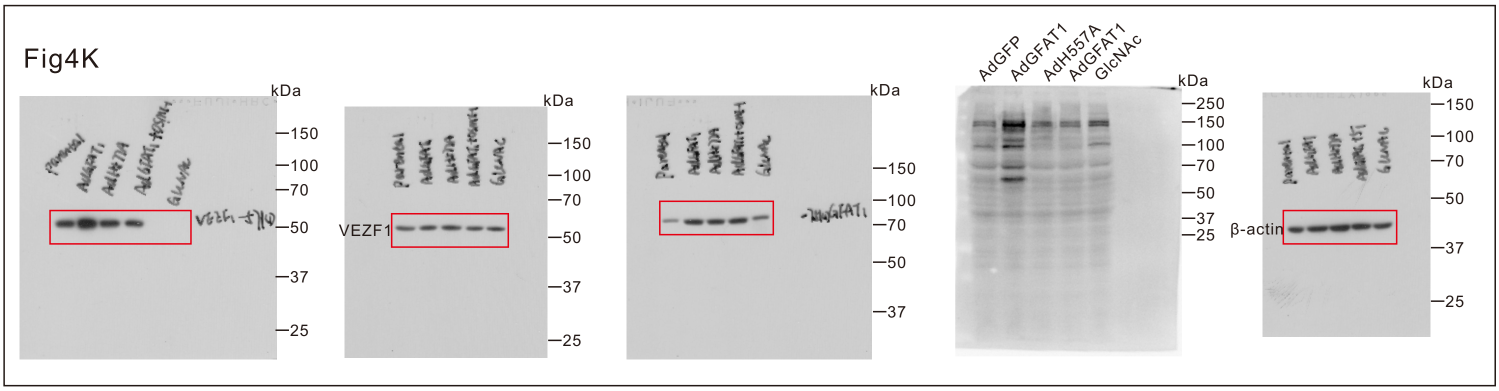

Fig4L

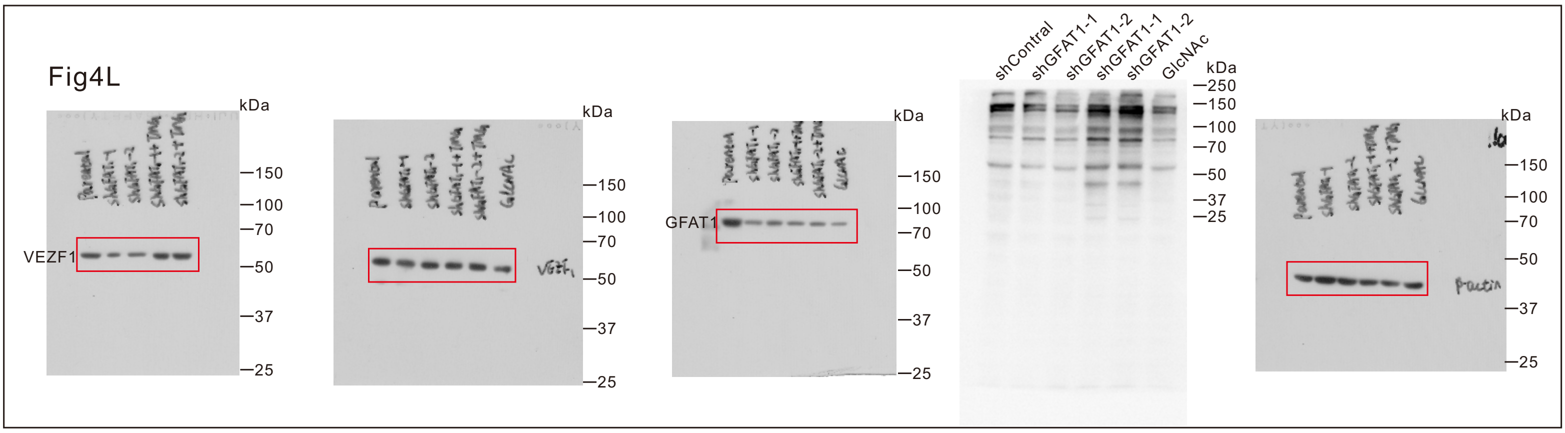

Fig5C

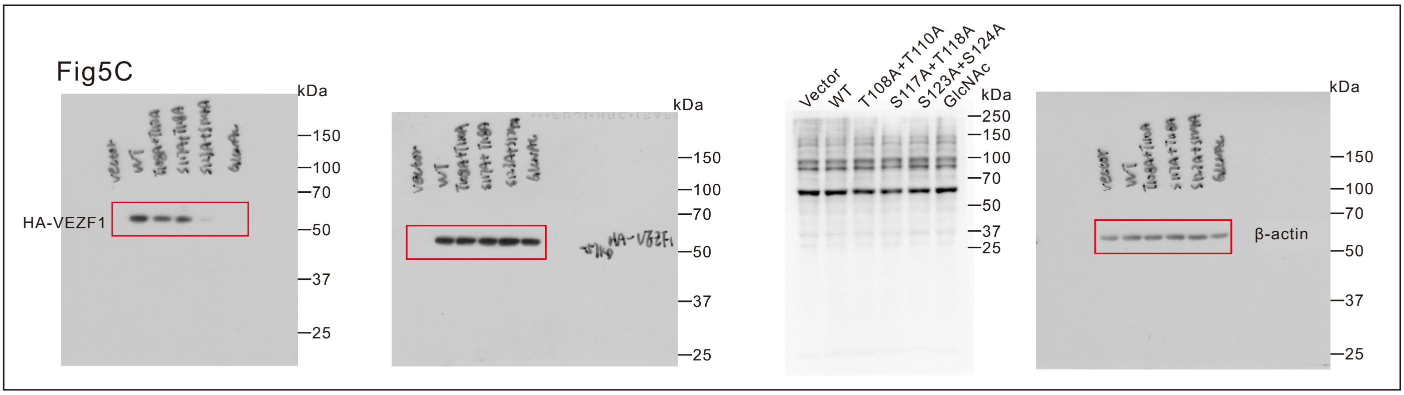

Fig5E

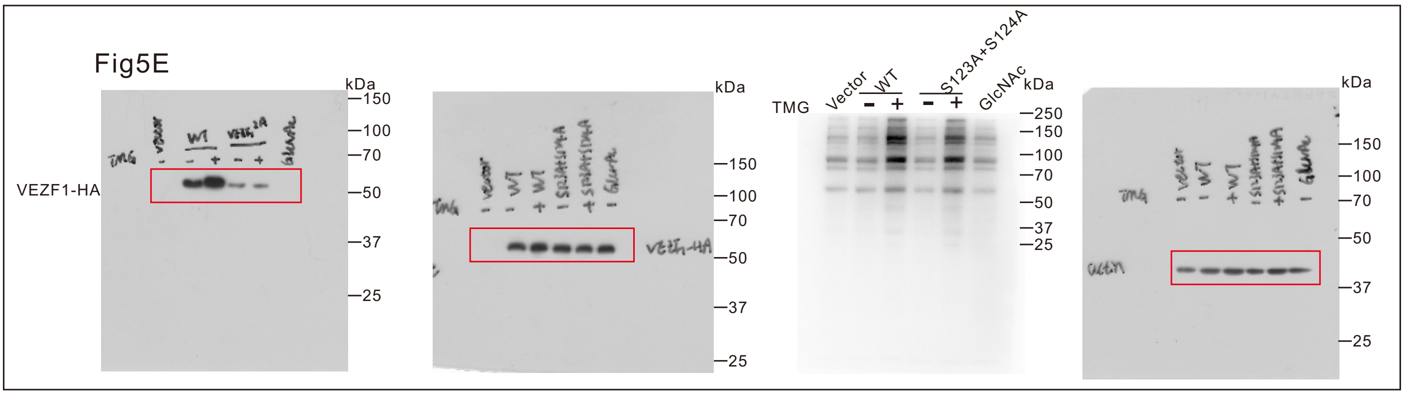

Fig5F

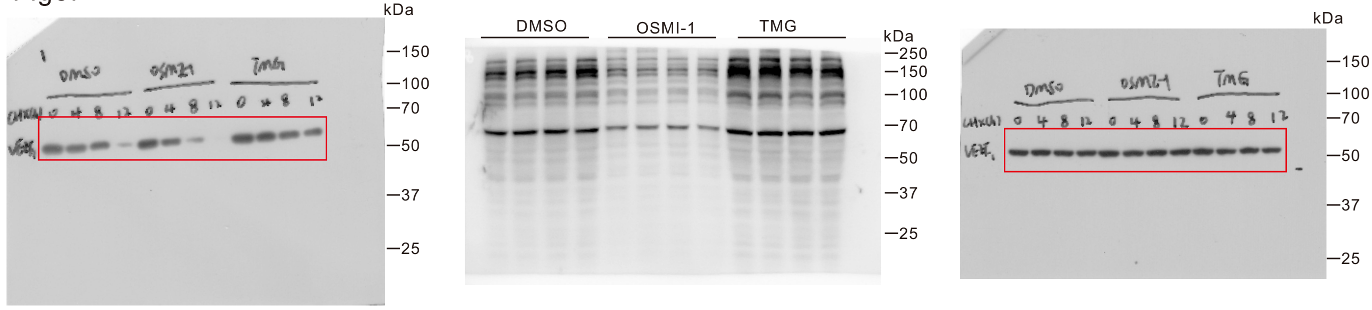

Fig5G

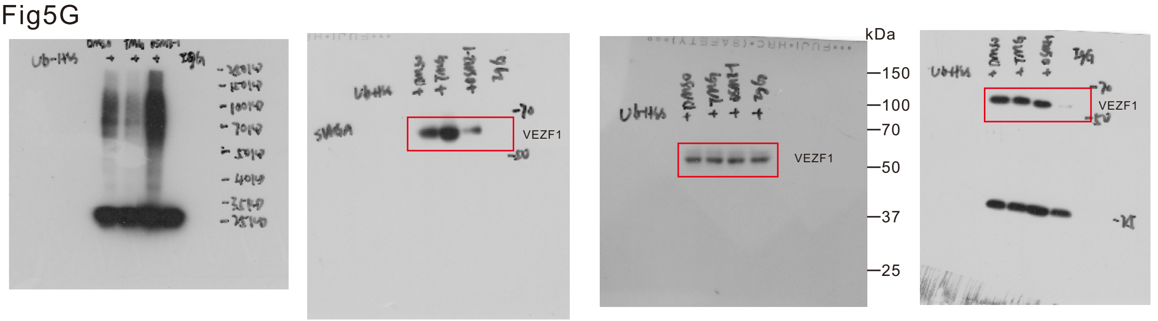

Fig5H

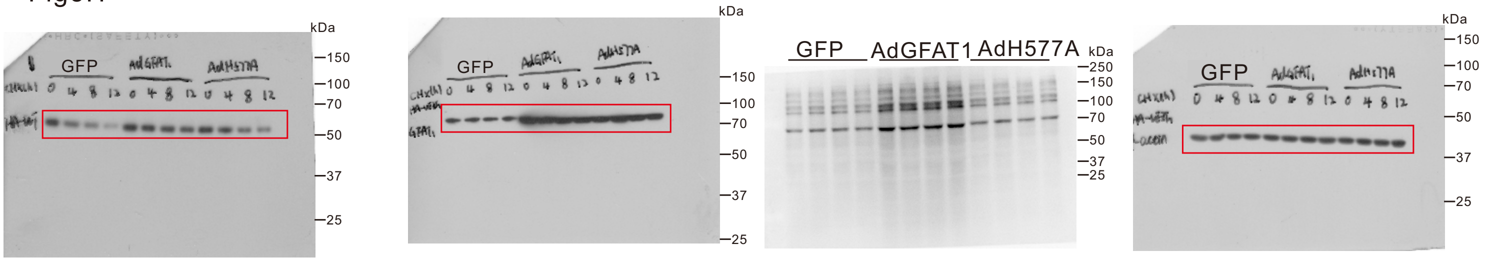

Fig5I

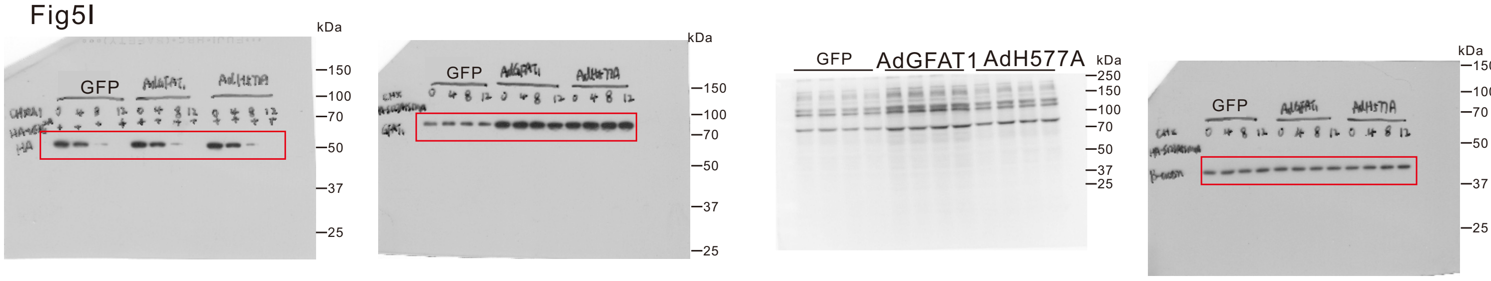

Fig5J

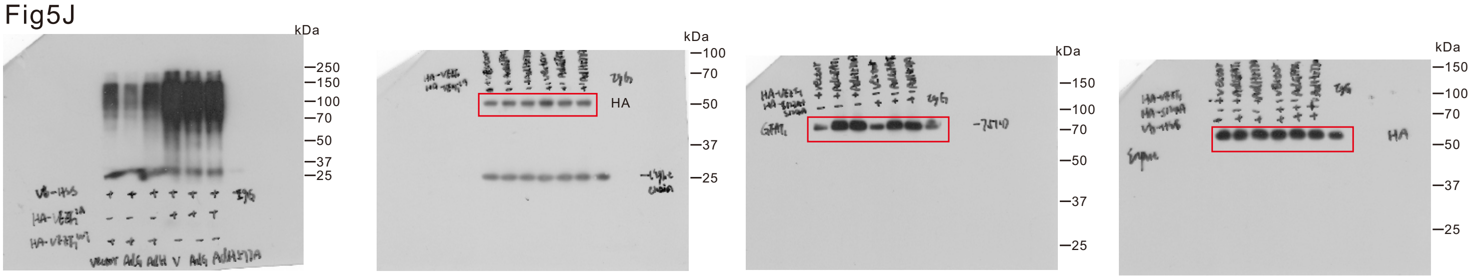

Fig6A

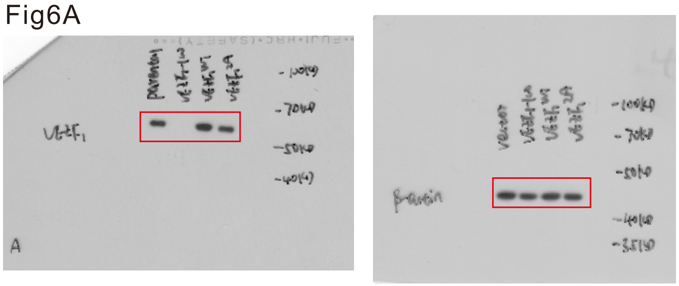

Fig6L

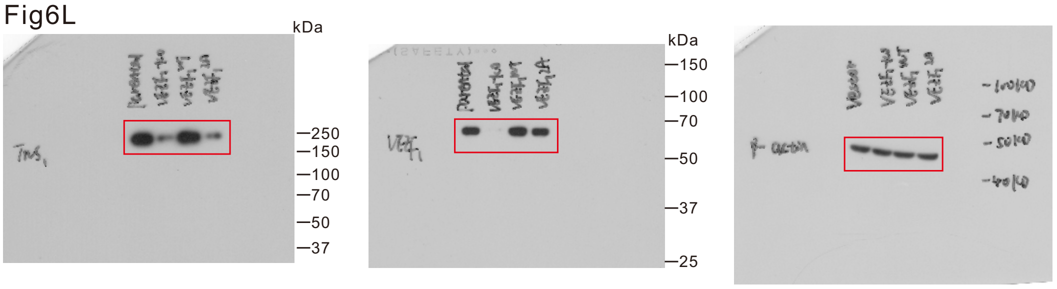

Fig6M

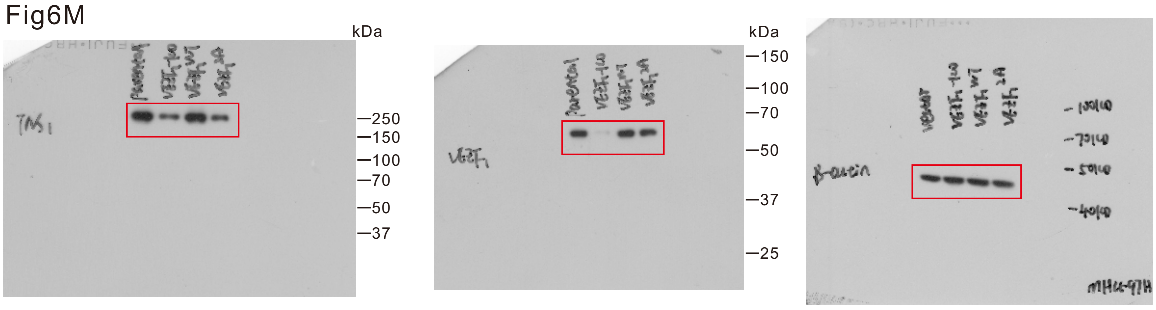

Fig7D

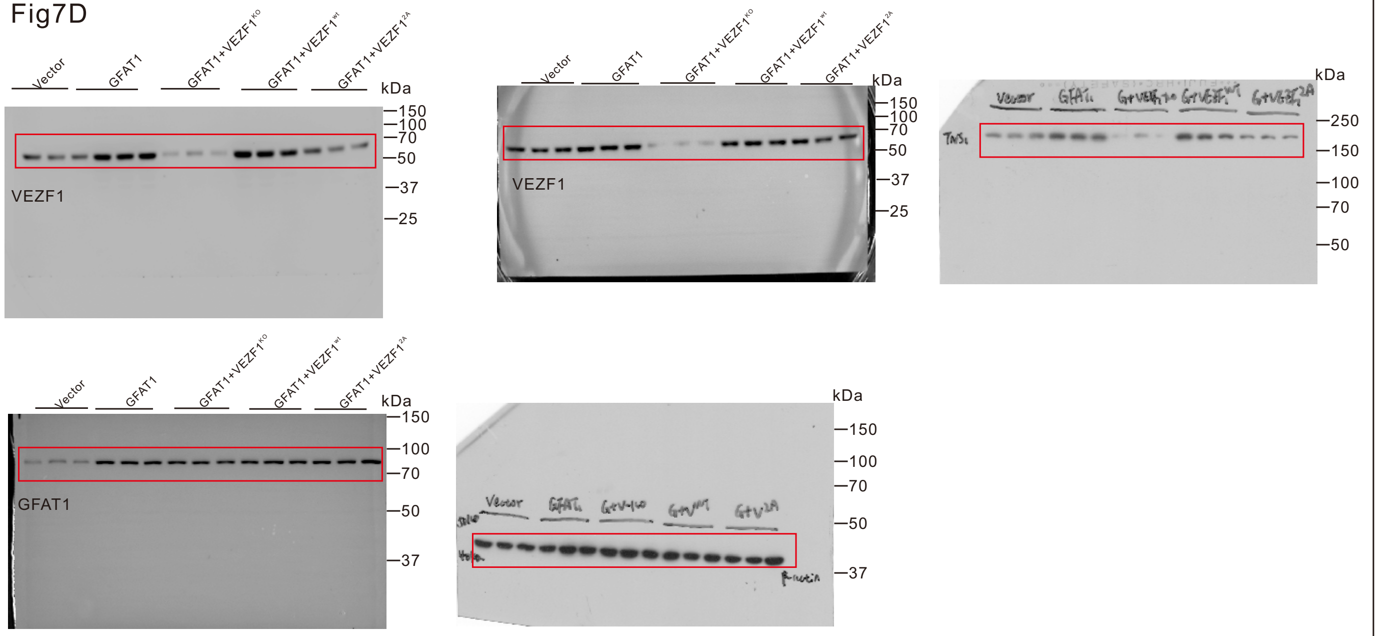

Fig8B

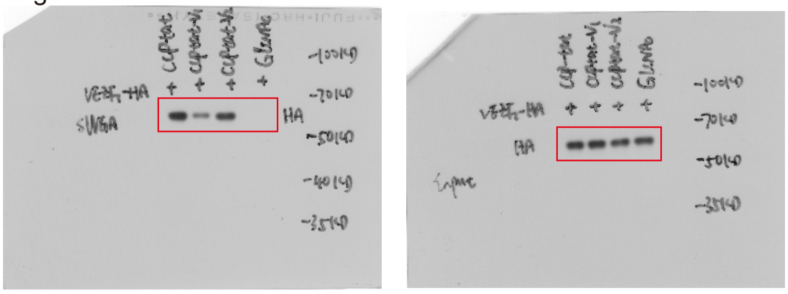

Fig8C

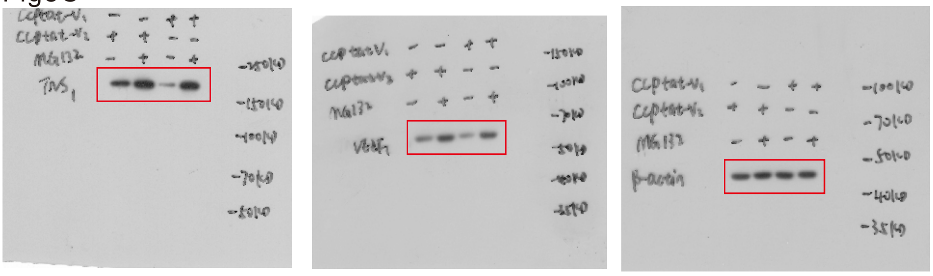

Fig8D

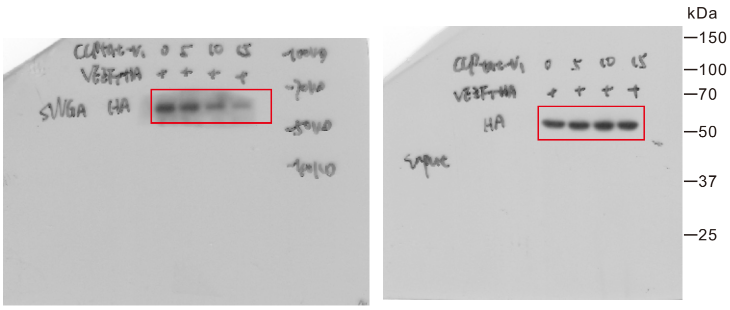

Fig8E

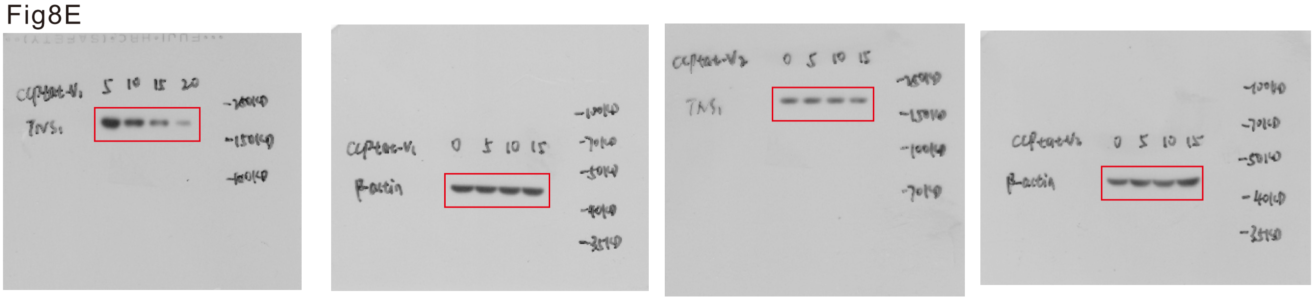

Fig8J

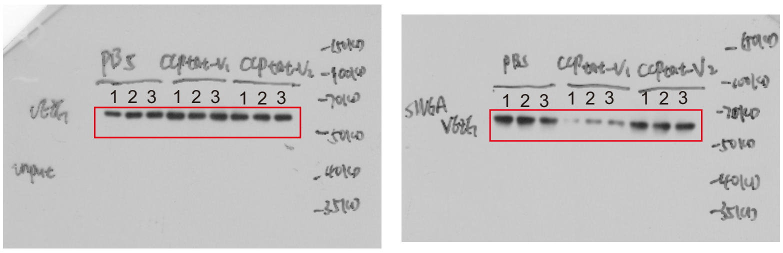

FigS1C

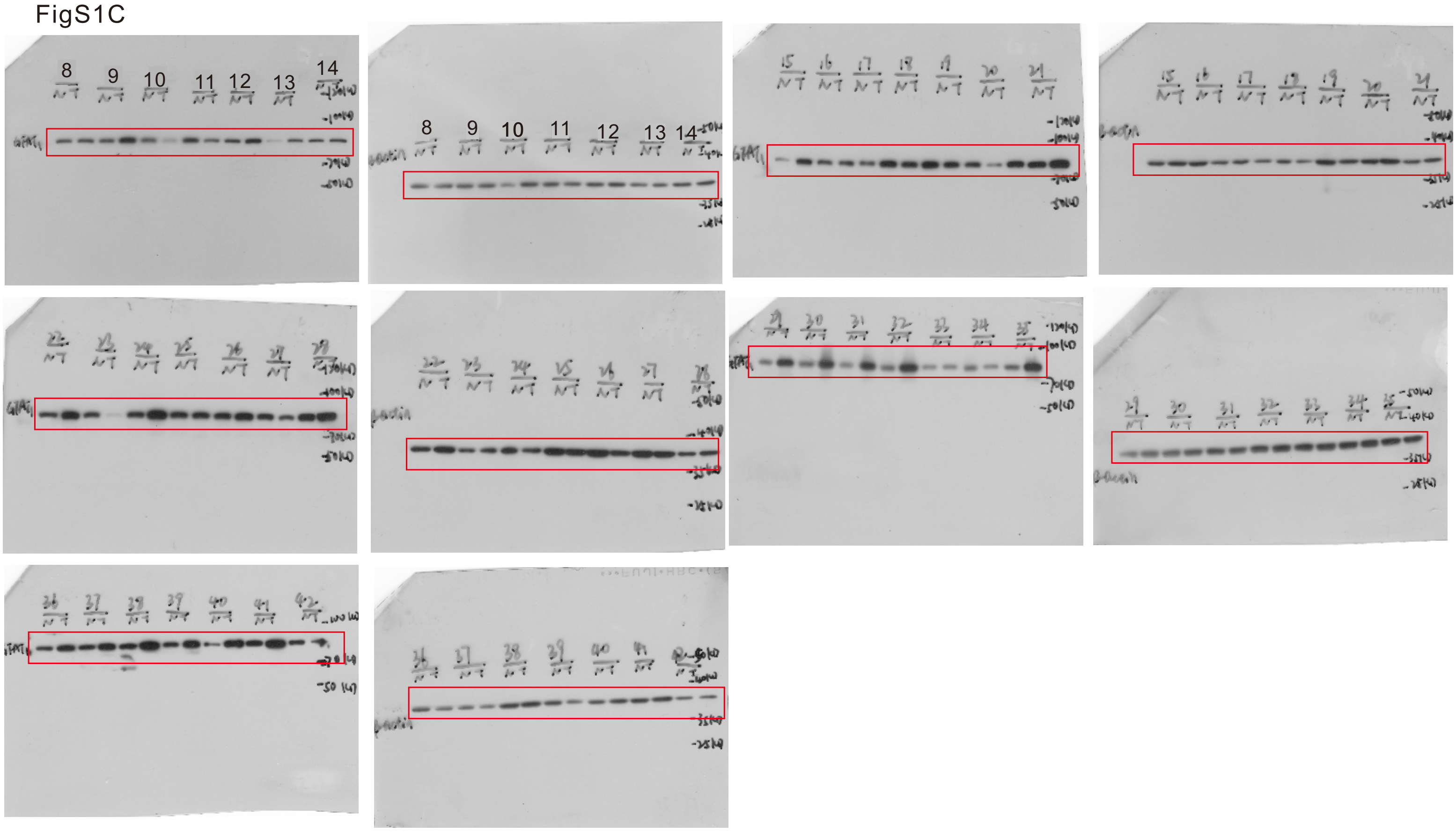

FigS2A

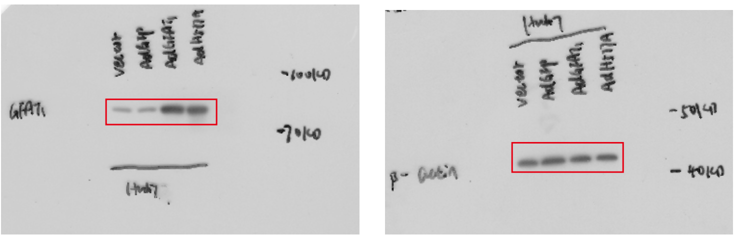

FigS4B

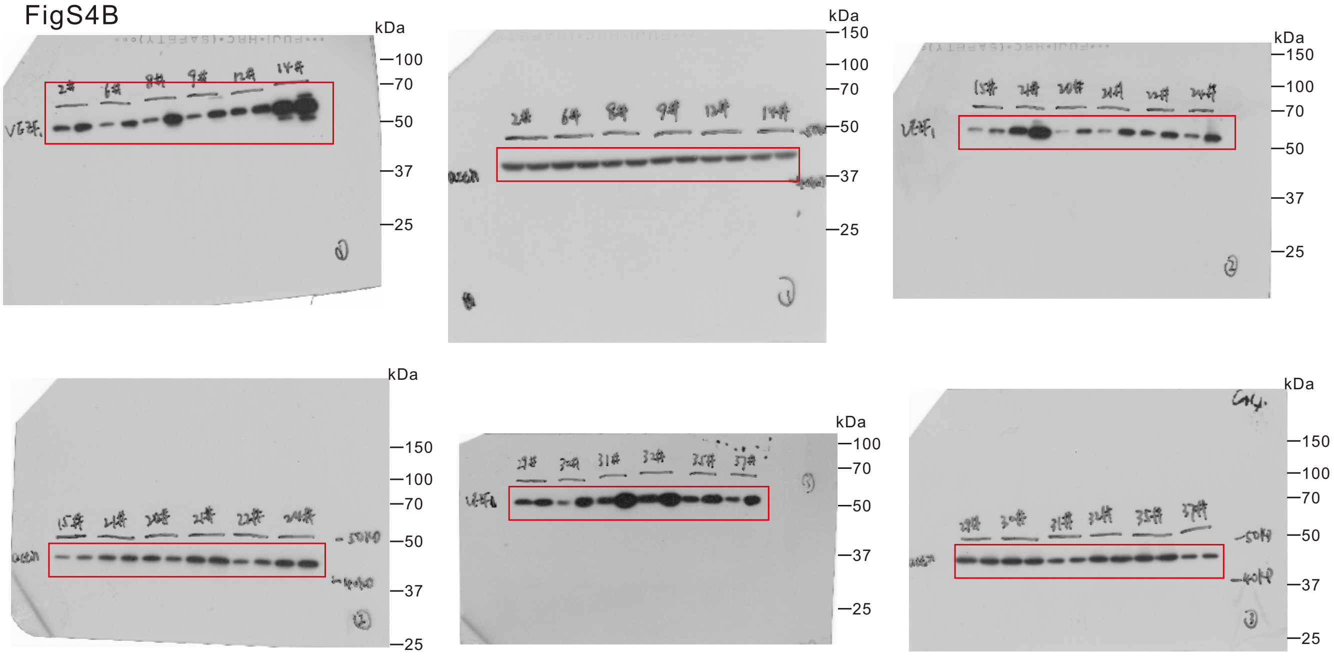

FigS5B

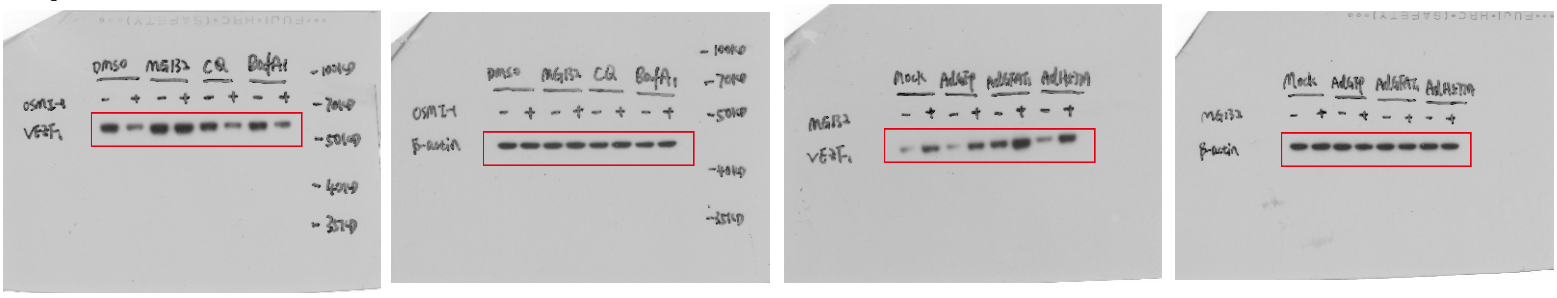

FigS5C

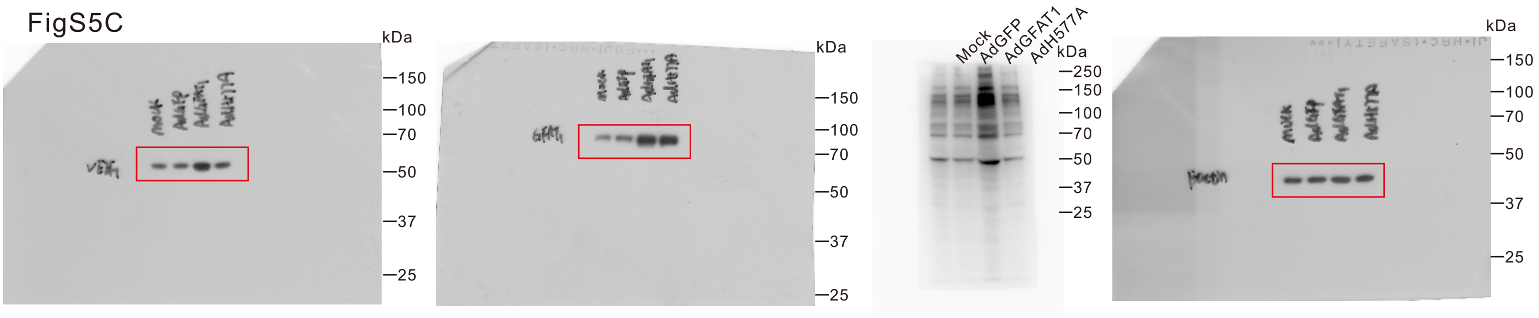

FigS6E

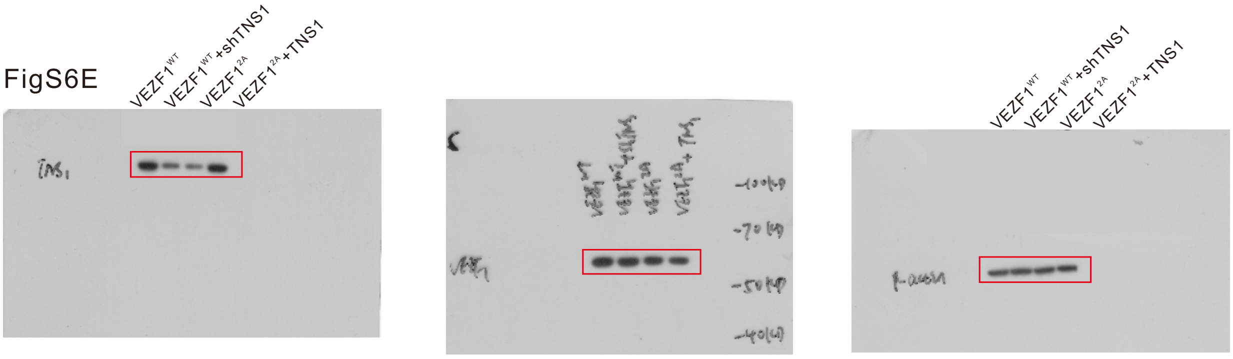

FigS7B

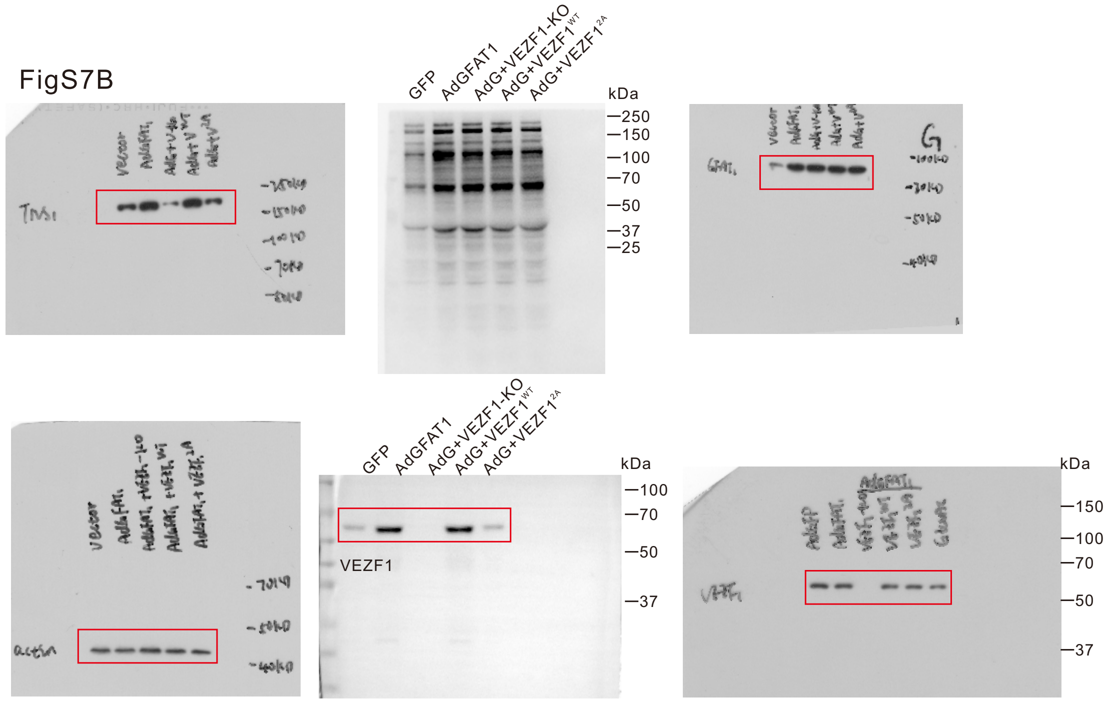

FigS7C

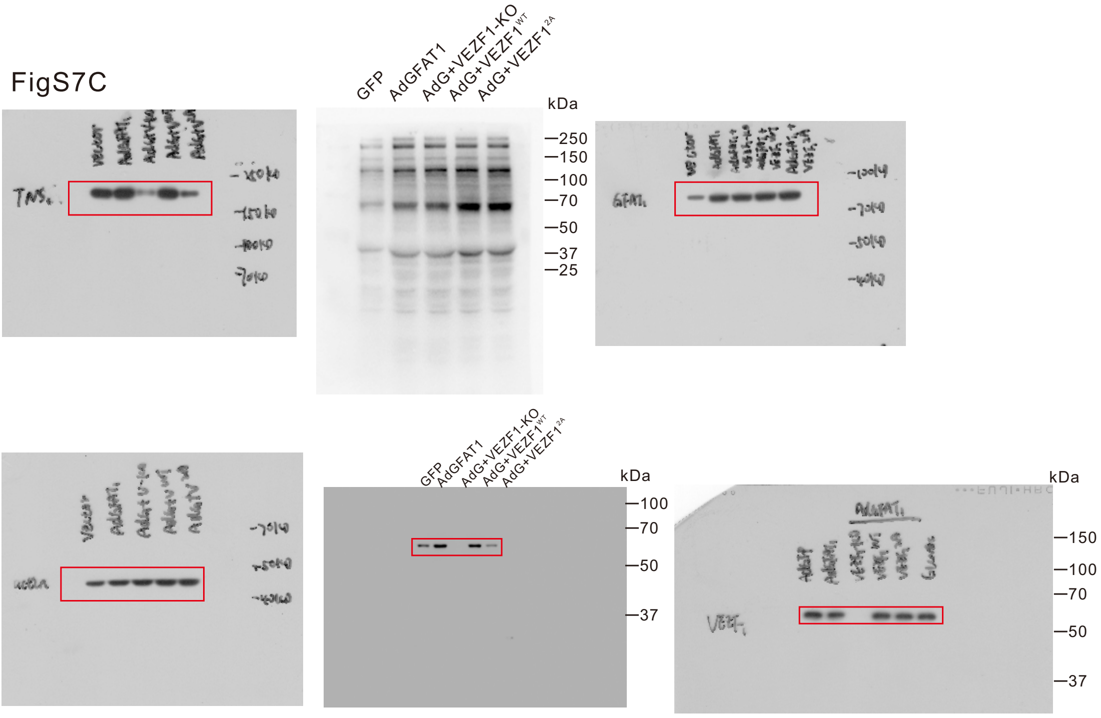

GFP
AdGFAT1
AdG+VEZF1-KO
AdG+VEZF1^WT
AdG+VEZF1^2A
kDa
250
150
100
70
50
37
25
kDa
100
70
50
37
kDa
150
100
70
50
37
